# Risks of covid-19 hospital admission and death for people with learning disability: population based cohort study using the OpenSAFELY platform

**DOI:** 10.1136/bmj.n1592

**Published:** 2021-07-15

**Authors:** Elizabeth J Williamson, Helen I McDonald, Krishnan Bhaskaran, Alex J Walker, Sebastian Bacon, Simon Davy, Anna Schultze, Laurie Tomlinson, Chris Bates, Mary Ramsay, Helen J Curtis, Harriet Forbes, Kevin Wing, Caroline Minassian, John Tazare, Caroline E Morton, Emily Nightingale, Amir Mehrkar, Dave Evans, Peter Inglesby, Brian MacKenna, Jonathan Cockburn, Christopher T Rentsch, Rohini Mathur, Angel Y S Wong, Rosalind M Eggo, William Hulme, Richard Croker, John Parry, Frank Hester, Sam Harper, Ian J Douglas, Stephen J W Evans, Liam Smeeth, Ben Goldacre, Hannah Kuper

**Affiliations:** 1 London School of Hygiene and Tropical Medicine, London, UK; 2 National Institute for Health Research (NIHR) Health Protection Research Unit in Vaccines and Immunisation, London, UK; 3 The DataLab, Nuffield Department of Primary Care Health Sciences, University of Oxford, Oxford, UK; 4 TPP, TPP House, Horsforth, Leeds, UK; 5 Public Health England, London, UK; 6 University of Bristol, Bristol, UK

## Abstract

**Objective:**

To assess the association between learning disability and risk of hospital admission and death from covid-19 in England among adults and children.

**Design:**

Population based cohort study on behalf of NHS England using the OpenSAFELY platform.

**Setting:**

Patient level data were obtained for more than 17 million people registered with a general practice in England that uses TPP software. Electronic health records were linked with death data from the Office for National Statistics and hospital admission data from NHS Secondary Uses Service.

**Participants:**

Adults (aged 16-105 years) and children (<16 years) from two cohorts: wave 1 (registered with a TPP practice as of 1 March 2020 and followed until 31 August 2020); and wave 2 (registered 1 September 2020 and followed until 8 February 2021). The main exposure group consisted of people on a general practice learning disability register; a subgroup was defined as those having profound or severe learning disability. People with Down’s syndrome and cerebral palsy were identified (whether or not they were on the learning disability register).

**Main outcome measure:**

Covid-19 related hospital admission and covid-19 related death. Non-covid-19 deaths were also explored.

**Results:**

For wave 1, 14 312 023 adults aged ≥16 years were included, and 90 307 (0.63%) were on the learning disability register. Among adults on the register, 538 (0.6%) had a covid-19 related hospital admission; there were 222 (0.25%) covid-19 related deaths and 602 (0.7%) non-covid deaths. Among adults not on the register, 29 781 (0.2%) had a covid-19 related hospital admission; there were 13 737 (0.1%) covid-19 related deaths and 69 837 (0.5%) non-covid deaths. Wave 1 hazard ratios for adults on the learning disability register (adjusted for age, sex, ethnicity, and geographical location) were 5.3 (95% confidence interval 4.9 to 5.8) for covid-19 related hospital admission and 8.2 (7.2 to 9.4) for covid-19 related death. Wave 2 produced similar estimates. Associations were stronger among those classified as having severe to profound learning disability, and among those in residential care. For both waves, Down’s syndrome and cerebral palsy were associated with increased hazards for both events; Down’s syndrome to a greater extent. Hazard ratios for non-covid deaths followed similar patterns with weaker associations. Similar patterns of increased relative risk were seen for children, but covid-19 related deaths and hospital admissions were rare, reflecting low event rates among children.

**Conclusions:**

People with learning disability have markedly increased risks of hospital admission and death from covid-19, over and above the risks observed for non-covid causes of death. Prompt access to covid-19 testing and healthcare is warranted for this vulnerable group, and prioritisation for covid-19 vaccination and other targeted preventive measures should be considered.

## Introduction

Identifying high risk groups for severe outcomes from covid-19 is critically important for risk stratification, which informs vaccine prioritisation initiatives and other targeted preventive measures. People with learning disability, who total more than one million people in England alone or 2% of the adult population, are one such vulnerable group.^1^ People with learning disability have a lower intellectual ability (usually IQ<70) and impaired social and adaptive functioning, with the onset in childhood. Learning disabilities are usually classified using a wide severity range (mild, moderate, severe, or profound), and consequently intensity of support needs differs widely.

As of February 2021, the Learning from Death Reviews programme reported that 1405 people with a learning disability had died from covid-19 in England since February 2020.^2^ The true number is probably far higher because of gaps in learning disability registration. The latest estimates from 2015 suggest that 23% of people with learning disability are included on the learning disability register.^1^ Emerging evidence from the first wave of the covid-19 pandemic in the United Kingdom showed that people with learning disability were at higher risk from mortality^3^ ^4^ ^5^ ^6^ ^7^ than people in the general population. For instance, the Oxford RCGP Research and Surveillance Centre sentinel network reported an odds ratio of 1.96 (95% confidence interval 1.22 to 3.18) for mortality during the first wave of infection in the UK among people with learning disability compared with those without.^4^ People with Down’s syndrome might be at particularly high risk; an analysis of primary care data from eight million adults reported a hazard ratio of 10.4 (7.1 to 15.2) for covid-19 death associated with Down’s syndrome.^8^ However, existing studies on the association of learning disability with severe outcomes from covid-19 do not include the second wave of the pandemic. Additionally, these studies frequently adjusted for variables that might be partly a consequence of the learning disability, such as deprivation and comorbidities, complicating interpretation of the results.^4^ A lack of clarity also exists on the increased risk of covid-19 deaths among people with milder learning disability, and this aspect needs exploration.^9^

The higher risk of premature death among people with learning disability in England is well known^1^ ^10^ and triggered the establishment of general practice learning disability registers to allow for better provision of their healthcare. A number of mechanisms exist which could increase the risk of covid-19 mortality in this group. People with learning disability have a higher prevalence of covid-19 mortality risk factors, including obesity, diabetes, epilepsy, and poverty.^11^ ^12^ ^13^ Medical conditions underlying the learning disability might confer additional risk; for instance, people with Down’s syndrome are more vulnerable to impaired cellular immunity, congenital heart disease, and respiratory conditions.^14^ ^15^ Many people with learning disability in England live in residential care or supported accommodation, or receive community based social care^16^; therefore, they have frequent contact with carers and other care recipients, and face challenges in physical distancing. Difficulties understanding the protective measures needed, compounded by a lack of accessible information, further increase the vulnerability of this group to infection.^17^ Healthcare access and quality, including prevention and treatment, are frequently worse for people with learning disability, leading to avoidable deaths.^10^ Treatment failures,^10^ including do not resuscitate orders,^18^ might increase their risk of death once infected.

Until 24 February 2021, the national recommendations for prioritisation of covid-19 vaccination in England included all adults with cerebral palsy, severe to profound learning disability, Down’s syndrome, and the whole resident population in care settings where a high proportion of residents would be eligible for vaccination (for example, due to learning disability).^19^ ^20^ This guidance means that not everyone on the learning disability register would be eligible for covid-19 vaccination, including people with mild to moderate learning disability from causes other than Down’s syndrome or cerebral palsy who are not living in residential care. This work was undertaken rapidly in response to an urgent need to inform policy making on vaccination prioritisation in the UK and elsewhere.

The aim of this study was to use linked electronic health records within the OpenSAFELY platform to rapidly describe the risk of covid-19 related hospital admissions and deaths among children and adults with learning disability in England compared with the general population. A subsidiary aim was to separate the risk by type of learning disability (severe to profound, cerebral palsy, Down’s syndrome, on the learning disability register), including people with learning disability not originally included in the first six groups of the phase 1 vaccination priority list in the UK.

## Methods

### Study design

We performed two population based, observational cohort studies of patients in England using data within the OpenSAFELY platform.

### Data

We used data from primary care linked to secondary care and mortality records in England. Records were linked to the NHS England inpatient activity datasets from Secondary Uses Service (SUS) data extracts, including data from inpatient activity datasets for determining covid-19 related hospital admissions.^21^ Office for National Statistics (ONS) death data were used to determine covid-19 related deaths. The dataset analysed within OpenSAFELY is based on 24 million people currently registered with general practice surgeries that use TPP SystmOne software—approximately 40% of the population in England. All data are pseudonymised and include coded diagnoses (using Read version 3, CTV3 codes), drugs, and physiological parameters. No free text data are included. The OpenSAFELY platform is a new data source and validation studies are not yet available. Other datasets based on primary care records in England have been shown to have high validity.^22^

### Study population

The first cohort comprised male and female patients (aged ≤105 years) registered as of 1 March 2020 in a general practice that uses the TPP system and followed until 31 August 2020. We excluded patients with missing age or a recorded age >105 years, missing sex, or missing postcode (from which much of the household and geographical information is calculated). Patients in the second cohort were similarly defined, but included those registered as of 1 September 2020 and followed until 8 February 2021. Patients in the two cohorts might differ slightly due to patients leaving and joining TPP practices, and patients dying prior to the start of the second cohort. These two time periods correspond to the two main waves of covid-19 infection experienced in England during 2020.^23^ In particular, 1 September 2020 had the lowest daily number of covid-19 related deaths since the start of the pandemic.^24^

### Exposures

All codelists used to define exposure groups are provided online, with links given in the supplementary appendix. The main exposure group comprised people on the learning disability register. This register contains a subset of people with learning disability; it is not a comprehensive list. However, the register provides a simple and practical means of identifying people for vaccine prioritisation or implementation of other public health measures. A subset of the codes used to define the learning disability register classified the learning disability as severe to profound, and were used to classify a subset of patients as having severe to profound learning disability.

Because a comprehensive indicator of residential care is lacking, those living in a household containing at least five people identified as being on the learning disability register were classed as being in residential care. Households were identified based on general practice registered addresses as of 1 February 2020, standardised and corrected using publicly available house sale data to remove registrations that are probably not current. We use the term residential care throughout, although we note that this includes a range of settings (care homes, educational settings, sheltered accommodation) and misclassification also probably exists. People with Down’s syndrome and cerebral palsy were identified based on general practice codes, which were reviewed by clinicians specialising in the care of people with learning disability (details in supplementary appendix).

### Outcomes

The outcomes for this study are covid-19 related death (defined as a covid-19 ICD-10 (international classification of diseases, 10th revision) code of U07.1 or U07.2 anywhere on the death certificate, determined from ONS death certificate data), and covid-19 related hospital admission (defined as admissions with any ICD-10 admission diagnosis, not restricted to primary diagnosis, of U07.1 or U07.2, determined from SUS data). People who had a covid-19 related hospital admission and then died contributed to both outcomes. An additional outcome of non-covid-19 death was also considered, determined from ONS death certificate data and excluding deaths classed as covid-19 related.

### Covariates

Covariates included demographics (age, sex, ethnicity, and geographical area), which could act as potential confounders, and current deprivation (index of multiple deprivation) as a potential mediator. To consider mediation by physical comorbidities that are also indications for vaccination we included body mass index ≥40, chronic cardiac disease, atrial fibrillation, deep vein thrombosis or pulmonary embolism, diabetes (further grouped by level of control, as measured by the latest glycated haemoglobin (HbA_1c_) measurement or lack of a measurement), chronic liver disease, stroke, transient ischaemic attack, dementia, asthma requiring use of oral corticosteroids, other chronic respiratory disease, reduced kidney function, dialysis, organ transplant, asplenia, other conditions leading to immunosuppression, and haematological cancer. We also included non-haematological cancer diagnosed in the past year, rheumatoid arthritis or lupus or psoriasis, and inflammatory bowel disease as common indications for immunosuppressing drugs. These conditions aim to map to the existing physical indications for vaccination among people aged 16-64 years in England; however data on epilepsy were not available for this analysis. We also identified other neurological conditions and serious mental illness to exclude people already prioritised for vaccination. We obtained these measures from medical records (details in supplementary appendix).

### Statistical methods

Analysis was undertaken separately for adults aged ≥16 years and children aged <16 years. We repeated analyses for the following exposures: being on the learning disability register (total, then divided into severe to profound *v* mild to moderate, and residential care *v* non-residential care), Down’s syndrome, and cerebral palsy.

The two cohorts for each wave were analysed separately. We used Cox proportional hazards for covid-19 related mortality and covid-19 related hospital admission, stratified by local geographical area as measured by the Sustainability and Transformation Partnership to account for differing patterns of infection over time in different regions, with days in study as the timescale. Follow-up was censored at competing events (non-covid death for mortality analyses and any cause death for hospital admission analyses) to target the cause specific hazard. Models adjusted for confounders (age, sex, ethnicity), additionally for deprivation, residential care status and physical comorbidities (described above), and then adjusted for all of these factors simultaneously. We explored exposure interactions with broad age ranges (16-64, 65-74, ≥75), sex, and deprivation. Tests based on Schoenfeld residuals were used to assess the proportional hazards assumption.

Similar Cox models were fitted for covid-19 related hospital admissions, after excluding people who were already prioritised for vaccination because of age or comorbidities as part of the first six priority groups of phase 1 in the UK. These were people aged ≥65 years and people with codes for physical conditions indicating priority for vaccination, other neurological conditions, severe mental illness, Down’s syndrome, cerebral palsy, and severe to profound learning disability. These Cox models adjusted for the confounders, then separately for deprivation and residential care status. Finally, Cox models for non-covid deaths were fitted, adjusting for the same variables as previous models.

For children <16 years, these analyses were undertaken separately. We omitted analyses looking at death, full adjustment for comorbidities, and interaction analyses because of smaller numbers of outcomes.

### Missing data

The main analysis took a complete case approach for missing ethnicity data (around 25% of records). Previous analyses using these data suggest the assumption required for complete case analysis for ethnicity—that missingness is unrelated to outcome given covariates—is approximately satisfied here.^12^

There were also missing data for body mass index, serum creatinine, and HbA_1c_ measurements. For each of these variables, previous research into their recording in UK primary care records suggested that multiple imputation would not be appropriate because the assumption that the data are missing at random is not met: for example, people who are underweight and overweight will be more likely to have their body mass index recorded in primary care^25^; serum creatinine measurement typically reflects monitoring because of underlying risk factors for chronic kidney disease or a known diagnosis^26^; and social disparities in monitoring of HbA_1c_ among people with diabetes.^27^ People with missing body mass index were assumed to be non-obese. People with no serum creatinine measurement were included in the category “no evidence of poor kidney function,” an approach which has been found to produce prevalence estimates of chronic kidney disease comparable to those from other sources.^28^ When categorising people with diabetes according to glycaemic control, those with no HbA_1c_ measurement were included in a separate group “diabetes, no Hba1c” as an indicator for diabetes with poorly monitored glycaemic control.

We undertook two sensitivity analyses. Firstly, multiple imputation was performed, creating 10 imputed datasets separately for adults and children, and imputing ethnicity using a multinomial regression model that included all covariates, outcome indicators, and a Nelson-Aalen estimate of the cumulative hazard in the imputation model. Estimates were combined by using Rubin’s rules. Secondly, a complete case analysis was performed discarding people with no body mass index measurement; when data are missing not at random, complete case analysis might be less biased than multiple imputation.^29^

### Software and reproducibility

The prespecified study protocol is archived with version control (https://github.com/opensafely/Published-Protocols/blob/master/Learning_Disability_Covid_Protocol_2021_10_02.pdf). All code and codelist data are shared openly for review and reuse under the MIT open license (https://github.com/opensafely/absolute-risks-covid-research, https://codelists.opensafely.org/). The OpenSAFELY framework guarantees that the analytic code, clinical codelists, data processing logic, and all dependent libraries remain available and executable against randomly generated dummy data. The full analysis can be rerun against real data with one click by any person with the necessary information governance approvals.

### Patient and public involvement

We have developed a publicly available website (https://opensafely.org/) through which we invite any patient or member of the public to contact us about this study or the broader OpenSAFELY project. We were not able to undertake extensive patient or public involvement during the short time frame for producing results. However, we did consult with three clinicians specialising in the care of people with learning disabilities. An online Health Protection Research Unit (HPRU) in Vaccines and Immunisation public engagement event in December 2020 discussing the use of pseudonymised medical records to investigate who is at higher risk of severe covid-19 found that this research approach was supported and expected as part of the pandemic response.

## Results

Table 1 shows the baseline characteristics of people included in the analysis, given separately for those aged ≥16 years and those aged <16 years (characteristics in wave 2 were similar). Figure A1 in the supplementary appendix shows a flowchart of participant numbers through the study selection process.

**Table 1:** Baseline characteristics of adults and children in main analysis (with complete ethnicity data). Data are numbers (%)

Characteristics	Adults (≥16 years)			Children (<16 years)	
	On learning disability register	Not on learning disability register		On learning disability register	Not on learning disability register
Total	90 307 (100)	14 221 716 (100)		9298 (100)	2 617 720 (100)
Age group					
0-15	0 (0)	0 (0)		9298 (100)	2 617 720 (100)
16-44	52 333 (58)	6 360 525 (45)		0 (0)	0 (0)
45-64	29 114 (32)	4 633 383 (33)		0 (0)	0 (0)
65-69	3870 (4)	889 199 (6)		0 (0)	0 (0)
70-74	2704 (3)	894 793 (6)		0 (0)	0 (0)
75-79	1371 (2)	622 787 (4)		0 (0)	0 (0)
≥80	915 (1)	821 029 (6)		0 (0)	0 (0)
Sex					
Female	36 801 (41)	7 356 253 (52)		3013 (32)	1 276 612 (49)
Male	53 506 (59)	6 865 463 (48)		6285 (68)	1 341 108 (51)
Ethnicity					
White	81 261 (90)	12 044 684 (85)		7120 (77)	2 071 494 (79)
Black	1710 (2)	402 235 (3)		379 (4)	96 181 (4)
South Asian	5499 (6)	1 179 377 (8)		1271 (14)	279 286 (11)
Mixed	1125 (1)	206 860 (1)		307 (3)	99 517 (4)
Other	712 (1)	388 560 (3)		221 (2)	71 242 (3)
Region					
East	18 479 (20)	3 280 536 (23)		1850 (20)	618 211 (24)
London	3619 (4)	1 166 888 (8)		565 (6)	167 329 (6)
Midlands	21 480 (24)	3 162 698 (22)		2202 (24)	609 609 (23)
North East, Yorkshire and The Humber	19 655 (22)	2 709 851 (19)		2071 (22)	493 405 (19)
North West	10 228 (11)	1 243 972 (9)		981 (11)	224 084 (9)
South East	5256 (6)	862 061 (6)		570 (6)	170 246 (7)
South West	11 590 (13)	1 795 710 (13)		1059 (11)	334 836 (13)
Index of multiple deprivation					
1 (least deprived)	9719 (11)	2 804 359 (20)		1239 (13)	486 452 (19)
2	13 589 (15)	2 850 388 (20)		1373 (15)	464 177 (18)
3	17 339 (19)	2 910 085 (20)		1648 (18)	492 125 (19)
4	21 635 (24)	2 877 452 (20)		2116 (23)	533 117 (20)
5 (most deprived)	28 025 (31)	2 779 432 (20)		2922 (31)	641 849 (25)
Learning disability and related					
Mild-moderate learning disability	74 116 (82)	—		7909 (85)	—
Severe-profound learning disability	16 191 (18)	—		1389 (15)	—
Not in residential care*	82 274 (91)	14 220 351 (100)		9235 (99)	2 617 669 (100)
Residential care*	8033 (9)	1365 (<1)		63 (1)	51 (<1)
No Down’s syndrome	83 179 (92)	14 220 854 (100)		8387 (90)	2 615 994 (100)
Down’s syndrome	7128 (8)	862 (<1)		911 (10)	1726 (<1)
No cerebral palsy	83 357 (92)	14 210 368 (100)		8789 (95)	2 613 598 (100)
Cerebral palsy	6950 (8)	11 348 (<1)		509 (5)	4122 (<1)
Comorbidities					
Body mass index≥40	5897 (7)	382 625 (3)		71 (1)	4412 (<1)
Asthma (with OCS use)	879 (1)	129 455 (1)		—	—
Cystic fibrosis	35 (<1)	3731 (<1)		—	—
Respiratory disease	3521 (4)	574 387 (4)		—	—
Chronic cardiac disease	5637 (6)	915 988 (6)		—	—
Atrial fibrillation	2311 (3)	507 890 (4)		—	—
Deep vein thrombosis or pulmonary embolism	2072 (2)	291 929 (2)		—	—
Diabetes					
With HbA_1c_ <58 mmol/mol	6793 (8)	855 216 (6)		—	—
With HbA_1c_ ≥58 mmol/mol	3417 (4)	401 014 (3)		—	—
With no recent HbA_1c_ measure	1324 (1)	160 068 (1)		—	—
Liver disease	459 (1)	85 620 (1)		—	—
Stroke	1966 (2)	287 098 (2)		—	—
Transient ischaemic attack	931 (1)	217 105 (2)		—	—
Dementia	1887 (2)	169 050 (1)		—	—
Other neurological disease	8857 (10)	129 745 (1)		—	—
Poor kidney function					
Stage 3a/3b, eGFR 30-60	2817 (3)	714 502 (5)		—	—
Stage 4/5, eGFR <30	518 (1)	68 534 (<1)		—	—
Dysplenia	141 (<1)	23 082 (<1)		—	—
Organ transplant	191 (<1)	12 990 (<1)		—	—
Conditions leading to immunosuppression	484 (1)	40 802 (<1)		—	—
Haematological malignancy					
Diagnosed in past year	25 (<1)	7138 (<1)		—	—
Diagnosed 2-5 years ago	80 (<1)	21 673 (<1)		—	—
Diagnosed >5 years ago	283 (<1)	49 569 (<1)		—	—
Cancer (non-haematological) in past year	196 (<1)	63 011 (<1)		—	—
RA/SLE/psoriasis	4277 (5)	710 575 (5)		—	—
Inflammatory bowel disease	871 (1)	178 869 (1)		—	—
Serious mental illness	8026 (9)	168 342 (1)		—	—

eGFR=estimated glomerular filtration rate; HbA_1c_=glycated haemoglobin; OCS=oral corticosteroid; RA=rheumatoid arthritis; SLE=systemic lupus erythematosus.

* Living in household containing at least five people identified as being on learning disability register.

Among 14 312 023 adults aged ≥16 years, 90 307 (0.63%) were identified as being on the learning disability register (table 1). The largest group consisted of people with mild to moderate learning disability (74 116, 82%). Additionally, 16 191 (18%) were identified as having severe to profound learning disability, and 8033 (9%) as being in residential care. In total, 7990 adults were identified as having Down’s syndrome, of whom 7128 (89%) were on the learning disability register; 18 298 adults were identified as having cerebral palsy, of whom 6950 (38%) were on the learning disability register. Those on the learning disability register were more likely to be male, younger, and living in more deprived areas. Comorbidities were similar across groups; more people with diabetes, obesity, other neurological disease, and diagnoses of serious mental illness were among those on the learning disability register.

Among 2 627 018 children aged <16 years (table 1), 9298 (0.35%) were identified as being on the learning disability register. Of the 2637 children identified as having Down’s syndrome, only 911 (35%) were on the learning disability register. Of the 4631 children identified as having cerebral palsy, 509 (11%) were on the learning disability register.

### Covid-19 related hospital admissions and deaths among adults ≥16 years

Adults aged ≥16 years were followed up for 7.1 million person years (mean 0.5 years per person) during wave 1 (1 March 2020-31 August 2020; 183 days). Among adults on the learning disability register, 538 (0.6%) had a covid-19 related hospital admission; there were 222 (0.25%) covid-19 related deaths and 602 (0.7%) non-covid deaths. Among adults not on the register, 29 781 (0.2%) had a covid-19 related hospital admission; there were 13 737 (0.1%) covid-19 related deaths and 69 837 (0.5%) non-covid deaths.

Adults were followed up for 6.3 million person years (mean 0.4 years per person) during wave 2 (1 September 2020-8 February 2021; 160 days). Among 91 358 adults on the learning disability register, 1004 (1.1%) had a covid-19 related hospital admission; there were 286 (0.3%) covid-19 related deaths and 524 (0.6%) non-covid deaths. Among 14 260 586 adults not on the register, 63 053 (0.4%) had a covid-19 related hospital admission; there were 19 778 (0.1%) covid-19 related deaths and 58 021 (0.4%) non-covid deaths.

**Fig 1:**
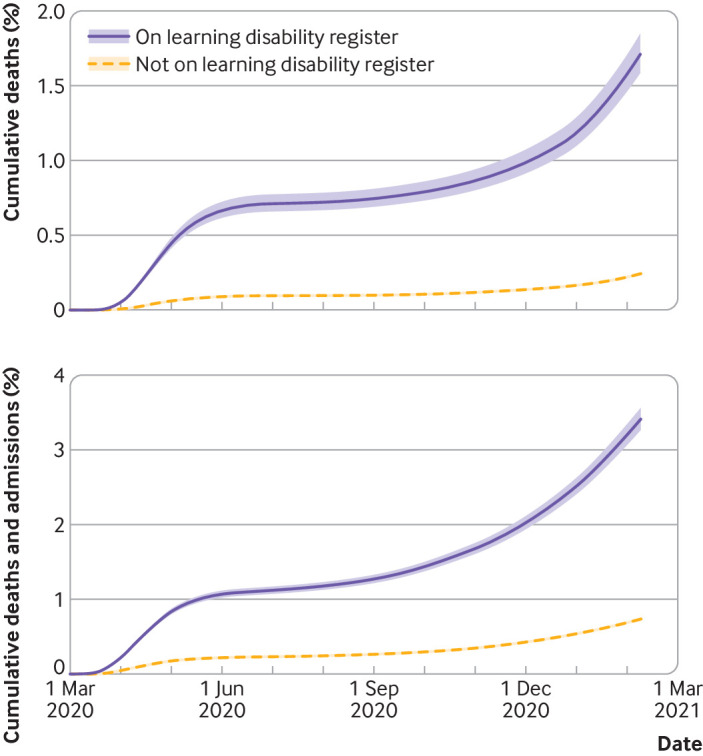
Cumulative covid-19 related deaths, and covid-19 related hospital admissions and deaths with 95% confidence intervals for adults aged ≥16 years through two waves of infection. People on learning disability register and those not on register shown separately. Estimates are standardised by age, sex, and ethnicity

Figure 1 shows cumulative covid-19 related deaths and hospital admissions among adults during the study period, accounting for sex, age, and ethnicity for people on the learning disability register and those who were not. Both graphs show a clear increase in events among those on the learning disability register, with a flattening off apparent during the period between waves of infection.

### Covid-19 related hospital admissions and deaths among children <16 years

Among children <16 years, 286 covid-19 related hospital admissions occurred in wave 1 during 1.3 million person years of follow-up (mean 0.5 years per person) and 529 in wave 2 during 1.1 million person years of follow-up (mean 0.4 years per person). Among 9298 children on the learning disability register during wave 1, there were five or fewer (≤0.05%) covid-19 related hospital admissions; we cannot provide the exact number because of stringent redaction rules applied to protect patient privacy. Among 9429 children on the learning disability register during wave 2, there were 20 (0.2%) covid-19 related hospital admissions. In total, across both waves, there were nine non-covid deaths among children on the register and 151 non-covid deaths among children not on the register. For covid-19 related deaths, there were five or fewer among children on the register and five or fewer among those not on the register. The number of deaths classed as covid-19 related was low among children overall.

### Hazard ratios for covid-19 hospital admissions and deaths among adults ≥16 years

For wave 1, the estimated hazard ratio for covid-19 related hospital admission among adults on the learning disability register (adjusted for age, sex, ethnicity, and geographical location) was 5.3 (95% confidence interval 4.9 to 5.8; table 2). The hazard ratio for covid-19 related death was 8.2 (7.2 to 9.4). Wave 2 produced similar estimates (4.3, 4.1 to 4.6 for covid-19 related hospital admission; 7.2, 6.4 to 8.1 for covid-19 related death). These associations were stronger among those with learning disability classed as severe to profound and among those in residential care. Down’s syndrome was associated with increased hazard of both events in both waves (wave 1: 10.6, 8.5 to 13.2 for covid-19 related hospital admission; 36.3, 26.7 to 49.5 for covid-19 related death; similar numbers were found for wave 2). Cerebral palsy was associated with higher hazards but to a lesser extent (wave 1: 5.0, 3.9 to 6.4 for covid-19 related hospital admission; 5.8, 4.1 to 8.3 for covid-19 related death; similar numbers were found for wave 2).

**Table 2:** Estimated hazard ratios for covid-19 outcomes among adults ≥16 years and children <16 years adjusted for potential confounders (age, sex, ethnicity, geographical region)

Exposure category	Covid-19 related hospital admission					Covid-19 related death			
	Events	Person years	Crude rate* (95% CI)	Hazard ratio (95% CI)		Events	Person years	Crude rate* (95% CI)	Hazard ratio (95% CI)
**Wave 1: adults (≥16 years)**									
Learning disability register									
No	29 781	7 133 803	4.2 (4.1 to 4.2)	Reference		13 737	7 140 988	1.9 (1.9 to 2.0)	Reference
Yes	538	45 126	11.9 (11.0 to 13.0)	5.30 (4.85 to 5.80)		222	45 257	4.9 (4.3 to 5.6)	8.21 (7.15 to 9.42)
Severity (learning disability register)									
Mild	391	37 070	10.5 (9.6 to 11.6)	4.74 (4.30 to 5.23)		159	37 167	4.3 (3.7 to 5.0)	7.15 (6.12 to 8.34)
Profound	147	8056	18.2 (15.5 to 21.4)	7.75 (6.43 to 9.33)		63	8090	7.8 (6.1 to 10.0)	13.14 (9.94 to 17.39)
Residential care status									
Not in residential care	438	41 141	10.6 (9.7 to 11.7)	4.87 (4.44 to 5.34)		182	41 249	4.4 (3.8 to 5.1)	7.81 (6.77 to 9.02)
In residential care	100	3984	25.1 (20.6 to 30.5)	8.72 (6.77 to 11.25)		40	4008	10.0 (7.3 to 13.6)	10.65 (7.08 to 16.03)
Down’s syndrome									
No	30 244	7 174 961	4.2 (4.2 to 4.3)	Reference		13 918	7 182 261	1.9 (1.9 to 2.0)	Reference
Yes	75	3968	18.9 (15.1 to 23.7)	10.59 (8.47 to 13.23)		41	3983	10.3 (7.6 to 14.0)	36.34 (26.67 to 49.51)
Cerebral palsy									
No	30 221	7 169 773	4.2 (4.2 to 4.3)	Reference		13 929	7 177 063	1.9 (1.9 to 2.0)	Reference
Yes	98	9156	10.7 (8.8 to 13.0)	4.95 (3.86 to 6.36)		30	9181	3.3 (2.3 to 4.7)	5.83 (4.12 to 8.26)
**Wave 1: children (<16 years)**									
Learning disability register									
No	>200†	1 318 599	0.2 (0.2 to 0.2)	Reference		—‡	—‡	—‡	—‡
Yes	≥5†	4700†	1.1 (0.4 to 2.6)	6.21 (2.75 to 14.05)		—‡	—‡	—‡	—‡
**Wave 2: adults (≥16 years)**									
Learning disability register									
No	63 053	6 262 945	10.1 (10.0 to 10.1)	Reference		19 778	6 270 857	10.1 (10.0 to 10.1)	Reference
Yes	1004	40 002	25.1 (23.6 to 26.7)	4.32 (4.05 to 4.61)		286	40 121	25.1 (23.6 to 26.7)	7.22 (6.41 to 8.13)
Severity (learning disability register)									
Mild	722	32 856	22 (20.4 to 23.6)	3.82 (3.55 to 4.11)		197	32 943	22.0 (20.4 to 23.6)	6.07 (5.29 to 6.96)
Profound	282	7146	39.5 (35.1 to 44.3)	6.52 (5.77 to 7.37)		89	7177	39.5 (35.1 to 44.3)	12.42 (10.06 to 15.33)
Residential care status									
Not in residential care	813	36 637	22.2 (20.7 to 23.8)	3.92 (3.64 to 4.21)		231	36 735	22.2 (20.7 to 23.8)	6.73 (5.91 to 7.66)
In residential care	191	3365	56.8 (49.3 to 65.4)	7.73 (6.51 to 9.19)		55	3385	56.8 (49.3 to 65.4)	10.37 (7.61 to 14.12)
Down’s syndrome									
No	63 898	6 299 465	10.1 (10.1 to 10.2)	Reference		19 998	6 307 480	10.1 (10.1 to 10.2)	Reference
Yes	159	3482	45.7 (39.1 to 53.3)	9.66 (8.28 to 11.27)		66	3498	45.7 (39.1 to 53.3)	38.50 (30.13 to 49.18)
Cerebral palsy									
No	63 865	6 294 857	10.1 (10.1 to 10.2)	Reference		20 030	6 302 862	10.1 (10.1 to 10.2)	Reference
Yes	192	8,090	23.7 (20.6 to 27.3)	4.23 (3.67 to 4.87)		34	8116	23.7 (20.6 to 27.3)	4.40 (3.14 to 6.17)
**Wave 2: children (<16 years)**									
Learning disability register									
No	509	1 137 827	0.4 (0.4 to 0.5)	Reference		—‡	—‡	—‡	—‡
Yes	20	4152	4.8 (3.1 to 7.5)	9.18 (5.89 to 14.29)		—‡	—‡	—‡	—‡

Wave 1: 1 March 2020-31 August 2020; wave 2: 1 September 2020-8 February 2021.

* Per 1000 person years.

† Rounded to avoid inadvertent disclosure of small event numbers. Median person years (25th-75th percentile): 0.504 (0.504 to 0.504) in wave 1 and 0.44 (0.44 to 0.44) in wave 2 for children and adults.

‡ Insufficient events for analysis.

Further adjustment for deprivation, residential care, and physical comorbidities only slightly attenuated these associations (supplementary appendix). For wave 1, the confounder adjusted hazard ratio for covid-19 related hospital admission for those on the learning disability register was 5.3 (95% confidence interval 4.9 to 5.8), reducing to 3.9 (3.6 to 4.3) after full adjustment. However, for Down’s syndrome, adjustment for residential care and comorbidities greatly attenuated hazard ratios (for example, the wave 1 hazard ratio for covid-19 related hospital admission reduced from 10.6 (8.5 to 13.2) to 6.4 (4.9 to 8.3) after adjustment for residential care, 7.2 (5.8 to 9.0) after adjustment for physical comorbidities, and 4.7 (3.6 to 6.0) after adjustment for all factors.

Tests of the proportional hazards assumption showed some evidence of non-proportionality (P values <0.001 for covid-19 related death and 0.176 for covid-19 related hospital admission in wave 1; corresponding values for wave 2 were 0.12 and 0.09, respectively). Therefore, the results reflect average hazard ratios over the follow-up periods.

### Hazard ratios for covid-19 hospital admission among children <16 years

Among children <16 years, being on the learning disability register was associated with increased hazard of covid-19 related hospital admission (wave 1: hazard ratio 6.2, 95% confidence interval 2.8 to 14.1; wave 2: 9.2, 5.9 to 14.3).

### Effect modification among adults ≥16 years

In wave 1, an interaction was observed between age and being on the learning disability register on the hazard of covid-19 related death: hazard ratio for being on the learning disability register 12.3, 95% confidence interval 10.0 to 15.1 for people aged 16-64; 10.5, 8.3 to 13.2 for people aged 65-74; and 4.2, 3.2 to 5.5 for people aged ≥75; similar estimates were observed for wave 2 (supplementary appendix). A similar interaction was not observed for covid-19 related hospital admission in waves 1 or 2. No interaction was observed by sex. Larger hazard ratios for covid-19 related hospital admission and death were observed for adults living in the least deprived areas compared with those in the more deprived areas, with similar patterns seen in both waves. Insufficient data were available to explore interactions by ethnicity.

### Hazard ratios among adults ≥16 years not prioritised for vaccination

After excluding people aged ≥65 years and those with defined comorbidities, the estimated hazard of covid-19 related hospital admission was 4.1 (95% confidence interval 3.3 to 5.2) after adjustment for age, sex, ethnicity, and geographical location, with little change after adjustment for deprivation or residential care status. Slightly attenuated associations were seen in wave 2 (3.0, 2.5 to 3.5) after adjustment for age, sex, ethnicity, and geographical location.

### Hazard ratios for non-covid death among adults ≥16 years

The estimated hazard ratio for non-covid death among adults (adjusted for age, sex, ethnicity, and geographical location) was 3.7 (95% confidence interval 3.4 to 4.0) in wave 1 and 4.0 (3.7 to 4.3) in wave 2. Associations were stronger among those classed as having severe to profound learning disability. Associations were slightly stronger among those classed as being in residential care. Down’s syndrome was associated with an increased hazard of non-covid death (wave 1: 12.3, 9.9 to 15.1; similar numbers were observed for wave 2). Cerebral palsy was associated with higher hazards to a lesser extent (wave 1: 3.2, 2.6 to 3.9 for covid-19 related death; similar numbers were observed for wave 2).

### Sensitivity analyses for missing data

Before excluding those with missing ethnicity data in wave 1, missing data occurred in 6 706 630 (28%) people for ethnicity, 7 027 371 (30%) for body mass index, and 221 283 (12% of 1 643 973 people with diabetes) for HbA_1c_. The measurement of serum creatinine typically reflects monitoring because of underlying risk factors for chronic kidney disease or a known diagnosis, reflected in approximately half of the sample (11 315 631, 48%) not having a serum creatinine measurement in the past five years. Wave 2 numbers were similar.

For adults, multiple imputation for missing ethnicity data made little difference to the results (supplementary appendix). When we analysed only people with recorded body mass index, the hazard ratios were slightly attenuated, but the pattern remained similar. For children, hazard ratios were slightly attenuated in wave 1 but similar in wave 2 when multiple imputation was performed to handle missing ethnicity data. Overall, our conclusions were robust to the method used to address missing data.

## Discussion

Our data show higher risk of hospital admission and death for all groups with learning disability compared with the general population. Generally, the pattern of hazard ratios is consistent for waves 1 and 2. For hospital admissions and deaths, slightly weaker associations were observed for wave 2 than for wave 1. Additionally, we found higher risks among those with severe to profound learning disability compared with those with mild to moderate learning disability, which was not explained by measured physical comorbidities or residential care status. However, the absolute number of deaths was higher among people with mild to moderate learning disability. 

For patients with Down’s syndrome or cerebral palsy, we observed higher risks among those on the learning disability register. Higher risks remained among those on the learning disability register who do not have Down’s syndrome or cerebral palsy compared with the general population. This observation is not explained by measured physical comorbidities or residential care status. After we excluded people who were prioritised for vaccination (first six groups of the phase 1 vaccination priority list in England due to age or comorbidities), those on the learning disability register still had a substantially increased risk of covid-19 related hospital admission. We also observed a higher risk of non-covid deaths in people with learning disability, though associations were weaker than for covid-19 related deaths. This finding is in contrast to most other risk factors that appear to have a similar magnitude of association with covid-19 related deaths and non-covid deaths.^30^

### Findings in context

Our findings are consistent with the existing literature, yet make an important contribution by including outcomes from the second wave of the pandemic and by showing the importance of the learning disability register in identifying people for vaccination prioritisation. The Oxford RCGP Research and Surveillance Centre sentinel network, which includes 4.4 million people who are nationally representative of the population in England, reported twofold higher mortality rates among people with learning disability (odds ratio 1.96, 95% confidence interval 1.22 to 3.18, P<0.01) after extensive adjustment.^4^ Public Health England used different sources of data and estimated that, up to June 2020, the mortality rate in people with learning disability was approximately 6.3-fold higher than that of the general population.^31^ Data from Scotland also showed that adults with intellectual disabilities had higher rates of covid-19 infection, severe infection, and mortality.^5^ Increased risks remained after adjusting for age, sex, and deprivation (standardised severe infection ratio: 2.59, 95% confidence interval 1.80 to 3.39; standardised mortality ratio 3.20, 2.1 to 4.25). Higher mortality rates among people with learning disability have also been reported in Wales,^6^ New York State,^7^ and the United States more broadly.^3^ Not only are death rates higher for people with learning disability, but deaths occur at younger ages.^3^ ^8^ ^9^ ^31^ The increased mortality rates among younger people with learning disability observed in our study have also been reported in the US.^3^ The younger age at death among people with learning disability is also well established for non-covid deaths.^10^

The high risk for people with Down’s syndrome was shown for the first wave by Clift and colleagues by using the QResearch population level primary care database^8^. In their cohort of eight million adults in England from January to June 2020, the age and sex adjusted hazard ratio for covid-19 death for adults with Down’s syndrome versus those without was 24.94 (95% confidence interval 17.08 to 36.44), which reduced to 10.39 (7.08 to 15.23) after extensive adjustment (deprivation, body mass index, cardiovascular, pulmonary and other disease, residential status, ethnicity). Data for cerebral palsy are more limited, but the QResearch analyses showed a higher mortality rate for this group (2.66, 1.62 to 4.36). Clift and colleagues obtained a fully adjusted hazard ratio of 1.27 (1.16 to 1.40) for covid-19 related death in those with learning disabilities other than Down’s syndrome, in contrast to our higher estimates. This discrepancy is probably because of differences in adjustment. We chose not to adjust for many comorbidities, viewing most of them as consequences of the learning disability and therefore part of the causal pathway. In our analyses, children with learning disability had a higher risk of hospital admission for covid-19. Existing studies also indicate that children with learning disability are more likely to require hospital admission and critical care because of covid-19 outcomes.^32^ ^33^ ^34^ ^35^ ^36^ However, it is important to note that the absolute risk of covid-19 related hospital admission experienced by children on the learning disability register was low.

While the incompleteness of the learning disability register means we will not have captured all deaths among children with learning disability, among children on the learning disability register there were fewer than five deaths per 1000 children years in each wave. Evidence about the relative risk of covid-19 related mortality associated with learning disability is lacking for children. The reason for this evidence gap is the small risk experienced by children overall—in our cohorts, few covid-19 related deaths occurred in children and so there were not enough data to analyse. Therefore, while we cannot quantify any differences in risk of covid-19 related deaths in children on the learning disability register, the absolute risk remains low.

The hazard ratios for covid-19 outcomes in our study attenuated after adjustment for deprivation. Residential status also partially explained the higher risks of severe covid-19 outcomes in people with learning disability. However, residential care for people with learning disability might not raise risk as much as in other care settings, perhaps because these facilities generally have fewer occupants.^31^ An important driver appears to be comorbidities, reflecting the higher prevalence of these covid-19 risk factors among people with learning disability.^11^ ^13^ Comorbidities are also an important driver of increased risk of non-covid deaths.^10^ However, large excess mortality rates remained after extensive adjustment, which is also apparent in previous studies.^8^ This pattern implies that other drivers might be relevant, including inherent clinical vulnerabilities for people with certain conditions and concerns about healthcare quality, as also indicated by the higher case fatality rates among people with learning disability (Scotland: 30% *v* 24%^13^; New York: 15.0% *v* 7.9%^7^; US 18-74 year olds: 4.5% *v* 2.7%^3^). Additional mechanisms might contribute to the high risk among people with Down’s syndrome, including impaired cellular immunity.^15^

### Strengths and weaknesses

Key strengths and weaknesses of the OpenSafely platform have been outlined previously.^12^ An important strength in the current analyses is that the study is large, including the records of approximately 40% of the English population, which allows disaggregation by learning disability grouping. We used comprehensive data on participants from medical records, which allowed us to adjust analyses successively to explore mechanisms for the association of learning disability and adverse covid-19 outcomes. Additionally, we were able to assess excess risks for both waves of the covid-19 pandemic, in terms of hospital admission and mortality outcomes. We also considered children and adults.

This study also has important limitations. Substantial geographical variation exists in the choice of electronic health record system,^37^ and so the population might not be fully nationally representative, although it is broadly representative in terms of ethnicity and deprivation.^12^ Identifying everyone with a learning disability from medical records alone was not possible. For instance, the most recent data, from 2015, suggest that 23% of people with learning disability are included on registers.^1^ The overall proportion of people on the learning disability register was 0.44% for England in 2015, slightly lower than the proportion in our study (0.63%); this potentially indicates an increase in coverage of the register in recent years. The reasons for under registration are unclear, but might include lack of perceived need and fear of stigma, among patients or clinicians. People on the learning disability register probably have more profound impairment and health conditions. For instance, in the current analyses 89% of people with Down’s syndrome were on the register and had higher mortality rates than people with Down’s syndrome who were not on the register. As a consequence, the hazard ratios might have been overestimated, although most of our sample had mild to moderate learning disability. Validation studies are not available for the Down’s syndrome and cerebral palsy codelists, although they were reviewed by clinicians specialising in the care of people with learning disability.

Our hospital admission data included only completed hospital admissions, therefore this outcome will have been under ascertained towards the end of the second wave. Defining hospital admission and death as covid-19 related depends on diagnosis, and validity of the outcome measure probably changed over time. If testing or clinical diagnosis of covid-19 differed for people with learning disability compared with the general population, this could have biased our estimates in either direction. Additionally, underlying health conditions could have been under ascertained in primary care records. Data were not available for epilepsy, which is a covid-19 risk factor and more common among people with learning disability.^11^ ^13^ We also had an incomplete, and probably underestimated, measure of residential care.^16^ ^38^

The consequences of do not resuscitate orders on the survival of people with learning disability have been raised as an area of concern, but we were not able to assess this impact in the current analyses. We did not have data on quality of treatment and so were not able to explore all our hypothesised pathways between learning disability and adverse covid-19 outcomes. Furthermore, these analyses focused only on severe covid-19 outcomes, and did not explore impacts on physical and mental health of people with learning disability, which probably occurred as a result of lockdown and other restrictions^32^ and require mitigation. We focused on the general population and so our analyses explored the combination of infection and severe outcomes once infected, therefore we were unable to disentangle associations with those two steps in the process. However, focusing on infected people only would induce biases due to unrepresentative testing.

### Policy implications and interpretation

In February 2021, the Joint Committee on Vaccination and Immunisation updated its guidance^36^ on which groups should be prioritised for vaccination. The existing priority groups, including those with Down’s syndrome, cerebral palsy or severe or profound learning disability, were extended to include all people on the learning disability register. This change was informed by a previous version of this analysis, which showed increased risk in people on the learning disability who were not already prioritised for vaccination. However, the current learning disability registers are incomplete,^1^ and updating them would help to inform prioritisation programmes. Relying on codelists as an alternative to learning disability registers is not pragmatic for prioritisation given the large number of codes that are non-specific for learning disability. Many other countries (eg, Germany^39^ and the US^40^) currently focus on prioritising people living in care homes and those with Down’s syndrome for vaccination, and should consider broadening this category to include other people with learning disability. Increased risks among children <16 years suggest that vaccination in this age group warrants further consideration. These data should also be used to inform future prioritisation for subsequent vaccination initiatives in England, and international vaccine prioritisation programmes. Besides vaccination, efforts should continue to protect people with learning disability from covid-19 adverse outcomes, including consideration of non-pharmacological interventions such as shielding, and ensuring adequate support to obtain prompt access to testing for covid-19 and to appropriate healthcare.

### Future research

The ONS data show that people with disabilities in general are at higher risk of covid-19 related mortality,^41^ but this outcome has not been explored through clinical databases, partly because of the complexity of generating codelists for broad ranges of conditions. However, using learning disability register data has highlighted the importance of public health surveillance and the need to develop indicators for disability. We used an indicator of care home residence to explore the extent to which this factor might mediate the association between learning disability and covid-19, but more detailed and accurate information on care home residence status is needed to understand how best to mitigate risk for people in residential care. More research is warranted on the excess covid-19 risks among people with Down’s syndrome. Cerebral palsy includes people with a broad range of conditions and severity, and a deeper exploration of covid-19 risk for this group is needed.

### Conclusion

People with learning disabilities have markedly increased risks of hospital admission and death from covid-19. Prompt access to covid-19 testing and healthcare is warranted for this group, and prioritisation for covid-19 vaccination and other targeted preventive measures should be considered.

**What is already known on this topic**

Emerging evidence has shown that people with learning disability are at higher risk from covid-19 related mortality compared with the general population
Existing studies on the association of learning disability with severe outcomes from covid-19 often adjusted for variables that that might be partly a consequence of the learning disability, such as deprivation and comorbidities, which complicates interpretation of results

**What this study adds**

Adults with learning disability and those with Down’s syndrome or cerebral palsy have markedly increased risks of hospital admission and death from covid-19
Similar patterns were observed for children, but absolute risks of covid-19 hospital admission and death were small
Prompt access to covid-19 testing and healthcare is warranted for this group, and prioritisation for covid-19 vaccination and other targeted preventive measures should be considered

## Data Availability

All data were linked, stored and analysed securely within the OpenSAFELY platform (https://opensafely.org/). Data include pseudonymised data such as coded diagnoses, drugs, and physiological parameters. No free text data are included. All code is shared openly for review and reuse under MIT open license (https://github.com/opensafely/absolute-risks-covid-research). Detailed pseudonymised patient data are potentially reidentifiable and therefore not shared. We rapidly delivered the OpenSAFELY data analysis platform without prior funding to deliver timely analyses on urgent research questions in the context of the global covid-19 health emergency: now that the platform is established we are developing a formal process for external users to request access in collaboration with NHS England; details of this process will be published shortly on https://opensafely.org/.

## Web extra

Extra material supplied by authors

Web appendix: Supplementary appendix

## Notes

Contributors: BG conceived the platform and the approach; BG and LS led the project overall and are guarantors. Contributions are as follows: design, EJW, HIM, HK, SJWE; data curation: CB, JP, JC, SH, SB, DE, PI, CEM; analysis, EJW, KB, AJW, CEM; funding acquisition: BG, LS; information governance: AM, BG, CB, JP; methodology: EJW, HIM, HK, SJWE, KB, AJW, BG, LS, CB, JP, JC, SH, SB, DE, PI, CEM; disease category conceptualisation and codelists: CEM, AJW, PI, SB, DE, CB, JC, JP, SH, HJC, KB, SB, AM, BM, LT, IJD, HIM, RM, HF; ethics approval: HJC, EJW, LS, BG; project administration: CEM, HJC, CB, SB, AM, LS, BG; resources: BG, LS, FH; software: SB, DE, PI, AJW, CEM, SD, CB, FH, JC, SH; supervision: BG, LS, SB; writing (original draft): EJW, HK, HIM, SJWE. All authors were involved in design and conceptual development and reviewed and approved the final manuscript. The corresponding author attests that all listed authors meet authorship criteria and that no others meeting the criteria have been omitted. LS, BG and HK are joint principal investigators; EJW and HIM are joint first authors.

Information governance: NHS England is the data controller; TPP is the data processor; and the key researchers on OpenSAFELY are acting on behalf of NHS England. This implementation of OpenSAFELY is hosted within the TPP environment which is accredited to the ISO 27001 information security standard and is NHS IG Toolkit compliant^42^ ^43^; patient data have been pseudonymised for analysis and linkage using industry standard cryptographic hashing techniques; all pseudonymised datasets transmitted for linkage onto OpenSAFELY are encrypted; access to the platform is through a virtual private network (VPN) connection, restricted to a small group of researchers; the researchers hold contracts with NHS England and only access the platform to initiate database queries and statistical models; all database activity is logged; only aggregate statistical outputs leave the platform environment following best practice for anonymisation of results such as statistical disclosure control for low cell counts.^44^ The OpenSAFELY research platform adheres to the obligations of the UK General Data Protection Regulation (GDPR) and the Data Protection Act 2018. In March 2020, the Secretary of State for Health and Social Care used powers under the UK Health Service (Control of Patient Information) Regulations 2002 (COPI) to require organisations to process confidential patient information for the purposes of protecting public health, providing healthcare services to the public and monitoring and managing the covid-19 outbreak and incidents of exposure; this sets aside the requirement for patient consent.^45^ Taken together, these provide the legal bases to link patient datasets on the OpenSAFELY platform. GP practices, from which the primary care data are obtained, are required to share relevant health information to support the public health response to the pandemic, and have been informed of the OpenSAFELY analytics platform.

The lead authors (BG and LS) affirm that the manuscript is an honest, accurate, and transparent account of the study being reported; that no important aspects of the study have been omitted; and that any discrepancies from the study as planned (and, if relevant, registered) have been explained.

Dissemination to participants and related patient and public communities: Dissemination of findings will be undertaken through a variety of routes. A lay summary and Easyread summary aimed at people with learning disability will be made available on the OpenSAFELY and HPRU in Vaccines and Immunisation websites (https://opensafely.org/ and https://immunisation.hpru.nihr.ac.uk/).

Provenance and peer review: Not commissioned; externally peer reviewed.
