# Supplementary material for: Risks of covid-19 hospital admission and death for people with learning disability: population based cohort study using the OpenSAFELY platform
Source: BMJ. 2021 Jul 15;374:n1592. doi: 10.1136/bmj.n1592 (PMC8278652; doi:10.1136/bmj.n1592)
Supplement: Supplementary file 1 — Web appendix: Supplementary appendix [file wile065251.ww.pdf]

Supplementary Appendix

for

OpenSAFELY: Risks of COVID-19 hospital admission and death for people with learning disability – a cohort study.

The OpenSAFELY Collaborative, Elizabeth J Williamson[1]*, Helen I McDonald[1,2]*, Krishnan Bhaskaran[1], Alex J Walker[3], Sebastian Bacon[3], Simon Davy[3], Anna Schultze[1], Laurie Tomlinson[1], Chris Bates[4], Mary Ramsay[2,5], Helen J Curtis[3], Harriet Forbes[6], Kevin Wing[1], Caroline Minassian[1], John Tazare[1], Caroline E Morton[3], Emily Nightingale[1], Amir Mehrkar[3], Dave Evans[3], Peter Inglesby[3], Brian MacKenna[3], Jonathan Cockburn[4], Christopher T Rentsch[1], Rohini Mathur[1], Angel YS Wong[1], Rosalind M Eggo[1], Will Hulme[3], Richard Croker[3], John Parry[4], Frank Hester[4], Sam Harper[4], Ian J Douglas[1], Stephen JW Evans[1], Liam Smeeth[1]†, Ben Goldacre[3]**†, Hannah Kuper[1]†

† Joint principal investigators; * joint first authors

[1] London School of Hygiene and Tropical Medicine, Keppel Street, London WC1E 7HT

[2] National Institute for Health Research Health Protection Research Unit in Vaccines and Immunisation, London, UK

[3] The DataLab, Nuffield Department of Primary Care Health Sciences, University of Oxford, OX26GG

[4] TPP, TPP House, 129 Low Lane, Horsforth, Leeds, LS18 5PX

[5] Public Health England, London.

[6] University of Bristol, Bristol, UK.

**Corresponding

Contents

- Figure A1. Flowchart showing patient numbers through the study selection process for each of the two cohorts (for the two waves)
- Table A1a. Estimated hazard ratio for COVID-19 related hospital admissions in wave 1 (1 March 2020 – 31 Aug 2020) in adults 16 and over
- Table A1b. Estimated hazard ratio for COVID-19 related death in wave 1 (1 March 2020 – 31 Aug 2020) in adults 16 and over
- Table A1c. Estimated hazard ratio for COVID-19 related hospital admissions in wave 2 (1 September 2020 – 8 February 2021) in adults 16 and over
- Table A1d. Estimated hazard ratio for COVID-19 related death in wave 2 (1 September 2020 – 8 February 2021) in adults 16 and over
- Table A2a. Estimated hazard ratio for COVID-19 related hospital admissions in wave 1 (1 March – 31st August 2020) in children under 16
- Table A2b. Estimated hazard ratio for COVID-19 related hospital admissions in wave 2 (1 September– 8 February 2021) in children under 16
- Table A3. Estimated hazard ratio for being on the learning disability register for COVID-19 related outcomes – interactions with age, sex and deprivation, adjusted for confounders and then all covariates
- Table A4. Estimated hazard ratio for being on the learning disability register for COVID-19 related hospital admission, after excluding those prioritised for vaccination through age or comorbidity in groups 1-6 of Phase I vaccination
- Table A5a. Estimated hazard ratio for non-COVID-19 related death in wave 1 (1 March – 31 August 2020) in adults 16 and over
- Table A5b. Estimated hazard ratio for non-COVID-19 related death in wave 2 (1 September 2020 – January 2021) in adults 16 and over
- Table A6. Baseline characteristics for adults and children among the whole sample, including those with missing ethnicity data
- Table A7a. Estimated hazard ratio for COVID-19 related death and hospital admissions in waves 1 (1 March 2020 – 31 August 2020) and 2 (1 September 2020 – January 2021) in adults 16 and over, comparing the main analysis to one handling missing data in BMI using a complete case approach and applying multiple imputation to handle missing ethnicity data
- Table A7b. Estimated hazard ratio for COVID-19 related hospital admissions waves 1 (1 March 2020 – 31 August 2020) and 2 (1 September 2020 – January 2021) in children under 16, comparing the main analysis with one using multiple imputation for missing ethnicity
- Table A8. Codelists used to define variables used in the analysis

Figure A1. Flowchart showing patient numbers through the study selection process for each of the two cohorts (for the two waves)

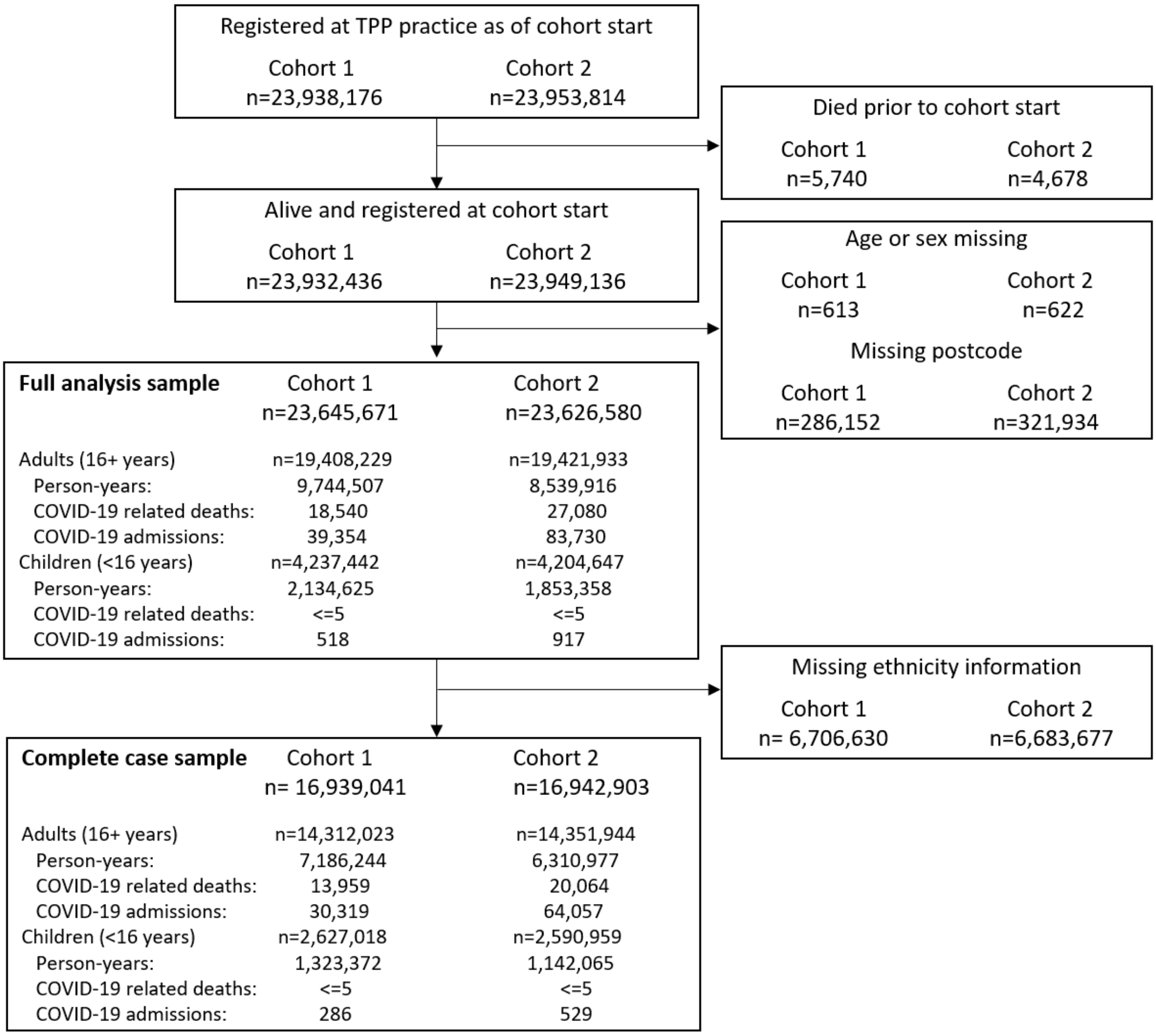

Table A1a. Estimated hazard ratio for COVID-19 related hospital admissions in wave 1 (1 March 2020 – 31 Aug 2020) in adults 16 and over

		Estimated Hazard Ratios (95% confidence intervals)				
Exposure category	*Events*	*Confounders*	*Confounders + IMD*	*Confounders + residential care*	*Confounders + comorbidities*	*All*
LDR						
No	29,781	Ref	Ref	Ref	Ref	Ref
Yes	538	5.30 (4.85, 5.80)	4.82 (4.41, 5.27)	4.70 (4.26, 5.18)	4.63 (4.24, 5.07)	3.92 (3.56, 4.30)
by severity:						
Mild-moderate	391	4.74 (4.3, 5.23)	4.27 (3.87, 4.72)	4.35 (3.91, 4.84)	4.08 (3.69, 4.5)	3.55 (3.19, 3.94)
Severe-profound	147	7.75 (6.43, 9.33)	7.27 (6.04, 8.76)	6.41 (5.3, 7.74)	7.28 (6.04, 8.79)	5.9 (4.89, 7.11)
by residential care status:						
Not in residential care	438	4.87 (4.44, 5.34)	4.41 (4.02, 4.84)		4.22 (3.85, 4.63)	3.98 (3.63, 4.37)
In residential care	100	8.72 (6.77, 11.25)	8.09 (6.27, 10.44)		8.08 (6.26, 10.44)	7.77 (6.01, 10.04)
Down's syndrome						
No	30,244	Ref	Ref	Ref	Ref	Ref
Yes	75	10.59 (8.47, 13.23)	10.23 (8.19, 12.79)	6.35 (4.88, 8.26)	7.22 (5.77, 9.03)	4.65 (3.59, 6.03)
Cerebral Palsy						
No	30,221	Ref	Ref	Ref	Ref	Ref
Yes	98	4.95 (3.86, 6.36)	4.65 (3.64, 5.95)	3.76 (2.93, 4.81)	4.86 (3.79, 6.22)	3.8 (2.98, 4.85)
Combined grouping						
None	29,734	Ref	Ref	Ref	Ref	Ref
DS but not LDR	[REDACTED]	4.18 (1.31, 13.33)	4.05 (1.27, 12.92)	4.16 (1.31, 13.22)	3.76 (1.17, 12.08)	3.65 (1.13, 11.72)
DS and LDR	[REDACTED]	11.52 (9.16, 14.49)	11.13 (8.84, 14.01)	9.91 (7.86, 12.51)	7.66 (6.07, 9.65)	6.53 (5.16, 8.26)
CP but not LDR	[REDACTED]	3.45 (2.47, 4.82)	3.25 (2.33, 4.52)	3.43 (2.45, 4.78)	3.4 (2.43, 4.76)	3.26 (2.33, 4.55)
CP and LDR	[REDACTED]	8.15 (5.95, 11.17)	7.65 (5.60, 10.44)	6.98 (5.13, 9.49)	7.92 (5.82, 10.79)	6.58 (4.87, 8.89)
LDR with no DS or CP	[REDACTED]	4.67 (4.24, 5.15)	4.21 (3.82, 4.65)	4.21 (3.78, 4.68)	4.14 (3.75, 4.57)	3.54 (3.19, 3.93)

LDR Learning Disability Register; DS Down's syndrome; CP Cerebral Palsy

Table A1b. Estimated hazard ratio for COVID-19 related death in wave 1 (1 March 2020 – 31 Aug 2020) in adults 16 and over

			Estimated Hazard Ratios (95% confidence intervals)			
Exposure category	*Events*	*Confounders*	*Confounders + IMD*	*Confounders + residential care*	*Confounders + comorbidities*	*All*
LDR						
No	13,737	Ref	Ref	Ref	Ref	Ref
Yes	222	8.21 (7.15, 9.42)	7.30 (6.35, 8.39)	6.96 (5.85, 8.28)	6.70 (5.81, 7.71)	5.58 (4.76, 6.53)
by severity:						
Mild-moderate	159	7.15 (6.12, 8.34)	6.32 (5.4, 7.4)	6.33 (5.27, 7.60)	5.76 (4.92, 6.76)	4.94 (4.15, 5.88)
Severe-profound	63	13.14 (9.94, 17.39)	12.02 (9.09, 15.89)	10.24 (7.44, 14.09)	11.35 (8.55, 15.07)	9.19 (6.8, 12.42)
by residential care status:						
Not in residential care	182	7.81 (6.77, 9.02)	6.93 (5.99, 8.01)		6.39 (5.52, 7.4)	5.96 (5.14, 6.9)
In residential care	40	10.65 (7.08, 16.03)	9.68 (6.44, 14.55)		8.53 (5.62, 12.95)	8.09 (5.32, 12.28)
Down's syndrome						
No	13,918	Ref	Ref	Ref	Ref	Ref
Yes	41	36.34 (26.67, 49.51)	34.21 (25.1, 46.64)	18.60 (12.64, 27.39)	16.23 (11.86, 22.22)	10.2 (7.08, 14.71)
Cerebral Palsy						
No	13,929	Ref	Ref	Ref	Ref	Ref
Yes	30	5.83 (4.12, 8.26)	5.43 (3.83, 7.7)	4.11 (2.8, 6.04)	5.7 (4.01, 8.09)	4.56 (3.17, 6.55)
Combined grouping						
None	13,721	Ref	Ref	Ref	Ref	Ref
DS but not LDR	[REDACTED]	15.07 (3.64, 62.32)	14.12 (3.39, 58.82)	14.98 (3.63, 61.74)	14.18 (3.38, 59.44)	13.44 (3.2, 56.52)
DS and LDR	[REDACTED]	39.63 (28.74, 54.64)	37.36 (27.09, 51.51)	32.08 (22.73, 45.27)	16.58 (11.94, 23.03)	13.97 (9.92, 19.69)
CP but not LDR	[REDACTED]	3.81 (2.29, 6.33)	3.57 (2.15, 5.92)	3.78 (2.27, 6.28)	3.7 (2.21, 6.18)	3.52 (2.11, 5.87)
CP and LDR	[REDACTED]	11.94 (7.42, 19.2)	11.02 (6.85, 17.74)	9.75 (5.88, 16.15)	11.88 (7.42, 19.03)	9.91 (6.08, 16.17)
LDR with no DS or CP	[REDACTED]	6.82 (5.86, 7.93)	6.04 (5.18, 7.03)	5.93 (4.94, 7.12)	5.72 (4.9, 6.67)	4.85 (4.1, 5.74)

LDR Learning Disability Register; DS Down's syndrome; CP Cerebral Palsy

Table A1c. Estimated hazard ratio for COVID-19 related hospital admissions in wave 2 (1 September 2020 – 8 February 2021) in adults 16 and over

		Estimated Hazard Ratios (95% confidence intervals)				
Exposure category	*Events*	*Confounders*	*Confounders + IMD*	*Confounders + residential care*	*Confounders + comorbidities*	*All*
LDR						
No	63,053	Ref	Ref	Ref	Ref	Ref
Yes	1,004	4.32 (4.05, 4.61)	3.91 (3.66, 4.18)	3.87 (3.59, 4.18)	3.8 (3.56, 4.05)	3.21 (2.98, 3.46)
by severity:	63,053					
Mild-moderate	722	3.82 (3.55, 4.11)	3.43 (3.19, 3.69)	3.54 (3.27, 3.83)	3.29 (3.06, 3.54)	2.87 (2.65, 3.1)
Severe-profound	282	6.52 (5.77, 7.37)	6.11 (5.4, 6.92)	5.50 (4.78, 6.33)	6.24 (5.53, 7.04)	5.08 (4.43, 5.82)
by residential care status:	63,053					
Not in residential care	813	3.92 (3.64, 4.21)	3.54 (3.27, 3.82)		3.41 (3.17, 3.66)	3.2 (2.97, 3.45)
In residential care	191	7.73 (6.51, 9.19)	7.17 (6.03, 8.52)		7.36 (6.21, 8.72)	7.06 (5.96, 8.36)
Down's syndrome						
No	63,898	Ref	Ref	Ref	Ref	Ref
Yes	159	9.66 (8.28, 11.27)	9.38 (8.05, 10.94)	6.49 (5.43, 7.76)	7.10 (6.09, 8.29)	5.00 (4.2, 5.95)
Cerebral Palsy						
No	63,865	Ref	Ref	Ref	Ref	Ref
Yes	192	4.23 (3.67, 4.87)	3.97 (3.45, 4.56)	3.33 (2.86, 3.87)	4.15 (3.61, 4.77)	3.28 (2.83, 3.8)
Combined grouping						
None	62,957	Ref	Ref	Ref	Ref	Ref
DS but not LDR	[REDACTED]	2.80 (1.26, 6.19)	2.73 (1.23, 6.05)	2.79 (1.26, 6.17)	2.53 (1.14, 5.61)	2.47 (1.11, 5.48)
DS and LDR	[REDACTED]	10.57 (9.03, 12.36)	10.26 (8.77, 11.99)	9.30 (7.93, 10.9)	7.61 (6.49, 8.91)	6.62 (5.64, 7.76)
CP but not LDR	[REDACTED]	3.15 (2.58, 3.85)	2.95 (2.42, 3.61)	3.13 (2.56, 3.82)	3.10 (2.54, 3.78)	2.95 (2.41, 3.59)
CP and LDR	[REDACTED]	6.44 (5.29, 7.84)	6.03 (4.96, 7.33)	5.57 (4.56, 6.81)	6.29 (5.17, 7.65)	5.21 (4.26, 6.37)
LDR with no DS or CP	[REDACTED]	3.73 (3.46, 4.01)	3.35 (3.1, 3.61)	3.39 (3.12, 3.69)	3.30 (3.07, 3.55)	2.82 (2.6, 3.06)

LDR Learning Disability Register; DS Down's syndrome; CP Cerebral Palsy

Table A1d. Estimated hazard ratio for COVID-19 related death in wave 2 (1 September 2020 – 8 February 2021) in adults 16 and over

		Estimated Hazard Ratios (95% confidence intervals)				
Exposure category	*Events*	*Confounders*	*Confounders + IMD*	*Confounders + residential care*	*Confounders + comorbidities*	*All*
LDR						
No	19,778	Ref	Ref	Ref	Ref	Ref
Yes	286	7.22 (6.41, 8.13)	6.39 (5.66, 7.22)	6.67 (5.86, 7.6)	6.31 (5.59, 7.11)	5.52 (4.85, 6.28)
by severity:	19,778					
Mild-moderate	197	6.07 (5.29, 6.96)	5.34 (4.64, 6.14)	5.79 (5.02, 6.67)	5.2 (4.52, 5.97)	4.65 (4.03, 5.37)
Severe-profound	89	12.42 (10.06, 15.33)	11.34 (9.16, 14.03)	11.26 (8.92, 14.2)	11.98 (9.75, 14.73)	10.69 (8.54, 13.39)
by residential care status:	19,778					
Not in residential care	231	6.73 (5.91, 7.66)	5.94 (5.2, 6.79)		5.86 (5.14, 6.69)	5.44 (4.76, 6.23)
In residential care	55	10.37 (7.61, 14.12)	9.39 (6.88, 12.82)		9.25 (6.84, 12.49)	8.77 (6.48, 11.87)
Down's syndrome						
No	19,998	Ref	Ref	Ref	Ref	Ref
Yes	66	38.5 (30.13, 49.18)	36.39 (28.46, 46.52)	25.88 (19.17, 34.94)	21.96 (17.23, 27.99)	16.17 (12.05, 21.7)
Cerebral Palsy						
No	20,030	Ref	Ref	Ref	Ref	Ref
Yes	34	4.4 (3.14, 6.17)	4.1 (2.93, 5.75)	3.34 (2.33, 4.79)	4.49 (3.2, 6.29)	3.62 (2.55, 5.15)
Combined grouping						
None	19,763	Ref	Ref	Ref	Ref	Ref
DS but not LDR	0	*	*	*	*	*
DS and LDR	65	44.68 (35, 57.04)	42.36 (33.19, 54.07)	41.08 (31.94, 52.84)	24.02 (18.8, 30.7)	22.37 (17.33, 28.87)
CP but not LDR	15	2.73 (1.68, 4.45)	2.55 (1.57, 4.16)	2.72 (1.67, 4.44)	2.76 (1.7, 4.48)	2.62 (1.61, 4.27)
CP and LDR	19	9.24 (5.77, 14.79)	8.55 (5.35, 13.65)	8.44 (5.21, 13.66)	9.77 (6.12, 15.6)	8.76 (5.44, 14.11)
LDR with no DS or CP	202	5.61 (4.86, 6.48)	4.94 (4.26, 5.74)	5.30 (4.55, 6.16)	4.98 (4.31, 5.77)	4.44 (3.82, 5.16)

LDR Learning Disability Register; DS Down's syndrome; CP Cerebral Palsy; * insufficient events

Table A2a. Estimated hazard ratio for COVID-19 related hospital admissions in wave 1 (1 March – 31st August 2020) in children under 16

	Estimated Hazard Ratios (95% confidence intervals)				
Exposure category	*Confounders*	*Confounders + IMD*	*Confounders + residential care*	*Confounders + comorbidities*	*All*
LDR					
No	Ref	Ref	Ref	Ref	Ref
Yes	6.21 (2.75, 14.05)	6.13 (2.71, 13.9)	6.24 (2.76, 14.12)	6.25 (2.76, 14.13)	6.20 (2.74, 14.05)
by severity:					
Mild-moderate	4.50 (1.59, 12.77)	4.44 (1.56, 12.63)	4.51 (1.59, 12.8)	4.53 (1.6, 12.83)	4.47 (1.57, 12.74)
Severe-profound	14.51 (3.52, 59.79)	14.47 (3.51, 59.69)	14.70 (3.57, 60.58)	14.62 (3.55, 60.31)	14.79 (3.58, 61.07)
by residential care status:					
Not in residential care	6.24 (2.76, 14.12)	6.16 (2.72, 13.97)		6.28 (2.78, 14.2)	6.20 (2.74, 14.05)
In residential care	*	*		*	*
Down's syndrome					
No	Ref	Ref	Ref	Ref	Ref
Yes	6.74 (1.67, 27.27)	6.74 (1.67, 27.26)	6.74 (1.67, 27.28)	6.76 (1.67, 27.37)	6.77 (1.67, 27.38)
Cerebral Palsy					
No	Ref	Ref	Ref	Ref	Ref
Yes	10.59 (4.2, 26.71)	10.52 (4.17, 26.51)	10.61 (4.21, 26.75)	10.61 (4.21, 26.75)	10.55 (4.19, 26.59)
Combined grouping					
None	Ref	Ref	Ref	Ref	Ref
DS but not LDR	4.77 (.67, 34.11)	4.79 (.67, 34.23)	4.77 (.67, 34.11)	4.77 (.67, 34.12)	4.79 (.67, 34.26)
DS and LDR	12.39 (1.74, 87.98)	12.27 (1.73, 87.11)	12.4 (1.75, 88.1)	12.52 (1.76, 88.98)	12.42 (1.75, 88.27)
CP but not LDR	9.14 (3.16, 26.49)	9.07 (3.14, 26.25)	9.14 (3.16, 26.48)	9.15 (3.16, 26.52)	9.08 (3.14, 26.28)
CP and LDR	23.87 (3.36, 169.75)	23.91 (3.37, 169.83)	24.18 (3.4, 171.99)	23.99 (3.37, 170.6)	24.36 (3.43, 173.07)
LDR with no DS or CP	4.48 (1.58, 12.68)	4.42 (1.55, 12.56)	4.5 (1.59, 12.74)	4.5 (1.59, 12.75)	4.46 (1.57, 12.68)

LDR Learning Disability Register; DS Down's syndrome; CP Cerebral Palsy; * insufficient events

Table A2b. Estimated hazard ratio for COVID-19 related hospital admissions in wave 2 (1 September– 8 February 2021) in children under 16

	Estimated Hazard Ratios (95% confidence intervals)				
Exposure category	*Confounders*	*Confounders + IMD*	*Confounders + residential care*	*Confounders + comorbidities*	*All*
LDR					
No	Ref	Ref	Ref	Ref	Ref
Yes	9.18 (5.89, 14.29)	8.98 (5.76, 13.98)	9.24 (5.93, 14.38)	8.96 (5.75, 13.97)	8.83 (5.67, 13.77)
by severity:					
Mild-moderate	7.18 (4.20, 12.27)	7.01 (4.1, 11.98)	7.21 (4.22, 12.32)	7.04 (4.13, 12.02)	6.92 (4.06, 11.81)
Severe-profound	19.04 (8.91, 40.71)	18.74 (8.76, 40.1)	19.37 (9.06, 41.41)	18.25 (8.48, 39.27)	18.27 (8.48, 39.35)
by residential care status:					
Not in residential care	9.24 (5.93, 14.38)	9.03 (5.8, 14.07)		9.02 (5.79, 14.05)	8.83 (5.67, 13.77)
In residential care	*	*		*	*
Down's syndrome					
No	Ref	Ref	Ref	Ref	Ref
Yes	16.63 (8.92, 31.0)	16.59 (8.90, 30.92)	16.64 (8.93, 31)	16.26 (8.74, 30.23)	16.25 (8.74, 30.21)
Cerebral Palsy					
No	Ref	Ref	Ref	Ref	Ref
Yes	14.49 (8.51, 24.66)	14.34 (8.43, 24.42)	14.51 (8.53, 24.7)	14.35 (8.43, 24.44)	14.24 (8.36, 24.25)
Combined grouping					
None	Ref	Ref	Ref	Ref	Ref
DS but not LDR	15.07 (6.74, 33.66)	14.99 (6.71, 33.48)	15.07 (6.75, 33.67)	14.97 (6.7, 33.47)	14.93 (6.68, 33.36)
DS and LDR	21.65 (7.98, 58.7)	21.66 (7.98, 58.77)	21.64 (7.98, 58.7)	20.71 (7.66, 55.94)	20.74 (7.67, 56.04)
CP but not LDR	12.35 (6.57, 23.2)	12.22 (6.51, 22.96)	12.35 (6.57, 23.2)	12.27 (6.53, 23.04)	12.14 (6.46, 22.8)
CP and LDR	24.79 (3.49, 176.18)	24.61 (3.46, 174.79)	25.12 (3.53, 178.59)	24.87 (3.50, 176.71)	24.99 (3.52, 177.54)
LDR with no DS or CP	3.09 (0.76, 12.59)	3.04 (0.74, 12.41)	3.1 (.76, 12.64)	3.10 (0.76, 12.66)	3.07 (0.75, 12.54)

LDR Learning Disability Register; DS Down's syndrome; CP Cerebral Palsy; * insufficient numbers to estimate.

Table A3. Estimated hazard ratio for being on the learning disability register for COVID-19 related outcomes – interactions with age, sex and deprivation, adjusted for confounders and then all covariates

Effect modifier	Wave 1 (1 March 2020 – 31 Aug 2020)		Wave 2 (September 2020 – 8 February 2021)	
	COVID-19 related hospital admission HR (95% CI)	COVID-19 death HR (95% CI)	COVID-19 related hospital admission HR (95% CI)	COVID-19 death HR (95% CI)
Confounder-adjusted models				
Age group				
16-64	5.37 (4.83, 5.97)	12.33 (10.04, 15.14)	4.15 (3.83, 4.5)	10.76 (9.02, 12.82)
65-74	6.59 (5.48, 7.92)	10.45 (8.28, 13.19)	5.67 (4.99, 6.45)	7.59 (6.14, 9.37)
≥75	3.52 (2.76, 4.48)	4.18 (3.19, 5.48)	3.35 (2.8, 4.00)	4.14 (3.23, 5.31)
Sex				
Male	6.10 (5.34, 6.97)	8.36 (6.62, 10.55)	4.98 (4.54, 5.47)	8.83 (7.42, 10.51)
Female	4.85 (4.32, 5.45)	8.12 (6.79, 9.72)	3.92 (3.60, 4.26)	6.28 (5.34, 7.39)
Index of Multiple deprivation				
1 (Least deprived)	7.31 (5.43, 9.84)	13.63 (8.97, 20.73)	6.89 (5.67, 8.37)	11.31 (8.01, 15.98)
2	6.08 (4.78, 7.74)	10.55 (7.61, 14.63)	5.67 (4.82, 6.68)	8.86 (6.44, 12.19)
3	5.63 (4.61, 6.87)	7.76 (5.69, 10.59)	4.72 (4.04, 5.52)	7.31 (5.57, 9.59)
4	5.12 (4.25, 6.16)	6.82 (5.08, 9.15)	3.46 (2.99, 4.00)	6.18 (4.90, 7.79)
5 (Most deprived)	3.57 (3.04, 4.20)	5.48 (4.21, 7.12)	3.01 (2.64, 3.44)	4.64 (3.65, 5.89)
Fully-adjusted models				
Age group				
16-64	3.91 (3.51, 4.35)	8.18 (6.62, 10.1)	3.05 (2.79, 3.32)	7.77 (6.48, 9.32)
65-74	4.70 (3.88, 5.69)	6.59 (5.16, 8.41)	4.14 (3.60, 4.75)	5.54 (4.48, 6.87)
≥75	2.87 (2.22, 3.70)	3.00 (2.22, 4.05)	2.73 (2.26, 3.29)	3.36 (2.60, 4.34)
Sex				
Male	4.22 (3.67, 4.86)	5.61 (4.34, 7.25)	3.46 (3.13, 3.82)	6.60 (5.50, 7.91)
Female	3.73 (3.31, 4.19)	5.56 (4.62, 6.69)	3.04 (2.77, 3.34)	4.89 (4.10, 5.83)
Index of Multiple deprivation				
1 (Least deprived)	5.34 (3.96, 7.2)	8.91 (5.73, 13.85)	5.04 (4.15, 6.12)	8.16 (5.80, 11.47)
2	4.42 (3.44, 5.67)	7.10 (4.97, 10.16)	4.23 (3.56, 5.03)	6.98 (5.08, 9.58)
3	4.36 (3.52, 5.39)	5.63 (4.00, 7.93)	3.72 (3.18, 4.35)	6.17 (4.72, 8.06)
4	4.23 (3.52, 5.07)	5.49 (4.09, 7.37)	2.86 (2.47, 3.30)	5.45 (4.30, 6.91)
5 (Most deprived)	3.15 (2.67, 3.70)	4.50 (3.44, 5.90)	2.66 (2.33, 3.05)	4.33 (3.40, 5.51)

Table A4. Estimated hazard ratio for being on the learning disability register for COVID-19 related hospital admission, after excluding those prioritised for vaccination through age or comorbidity in groups 1-6 of Phase I vaccination

	Estimated Hazard Ratios for COVID-19 hospital admissions (95% confidence intervals)		
	Confounders	*With IMD*	*With Residential care*
Wave 1 (1 March 2020 – 31 August 2020)	4.13 (3.31, 5.16)	3.91 (3.13, 4.89)	4.00 (3.17, 5.06)
Wave 2 (September 2020 – 8 February 2021)	2.96 (2.53, 3.47)	2.76 (2.36, 3.23)	2.63 (2.21, 3.13)

Table A5a. Estimated hazard ratio for non-COVID-19 related death in wave 1 (1 March – 31 August 2020) in adults 16 and over

		Estimated Hazard Ratios (95% confidence intervals)				
Exposure category	*Events*	*Confounders*	*Confounders + IMD*	*Confounders + residential care*	*Confounders + comorbidities*	*All*
LDR						
No	69,837					
Yes	602	3.69 (3.41, 4.00)	3.36 (3.08, 3.65)	3.3 (2.98, 3.65)	3.44 (3.17, 3.73)	2.97 (2.71, 3.27)
by severity:						
Mild-moderate	429	3.19 (2.91, 3.51)	2.89 (2.62, 3.18)	2.95 (2.65, 3.29)	2.94 (2.68, 3.23)	2.6 (2.35, 2.88)
Severe-profound	173	6.03 (5.18, 7.01)	5.62 (4.83, 6.55)	5.1 (4.25, 6.12)	5.91 (5.07, 6.88)	5.07 (4.27, 6.01)
by residential care status:						
Not in residential care	498	3.61 (3.30, 3.94)	3.28 (2.99, 3.59)		3.34 (3.05, 3.64)	3.12 (2.85, 3.41)
In residential care	98	4.35 (3.54, 5.35)	4.04 (3.27, 4.98)		4.09 (3.32, 5.05)	1.78 (1.19, 2.64)
Down's syndrome						
No	70,338					
Yes	101	12.26 (9.93, 15.13)	11.78 (9.52, 14.57)	8.62 (6.64, 11.18)	8.01 (6.44, 9.97)	6.15 (4.77, 7.94)
Cerebral Palsy						
No	70,341					
Yes	98	3.15 (2.56, 3.88)	2.97 (2.40, 3.68)	2.67 (2.14, 3.34)	3.12 (2.53, 3.85)	2.72 (2.19, 3.37)
Combined grouping						
None	69,787					
DS but not LDR	[REDACTED]	2.21 (0.54, 9.07)	2.10 (0.51, 8.66)	2.2 (.54, 9.03)	2.21 (.54, 9.08)	2.11 (0.51, 8.66)
DS and LDR	[REDACTED]	13.61 (10.92, 16.96)	13.09 (10.49, 16.33)	11.82 (9.27, 15.07)	8.53 (6.77, 10.76)	7.63 (5.96, 9.77)
CP but not LDR	[REDACTED]	2.19 (1.61, 2.97)	2.07 (1.52, 2.82)	2.18 (1.61, 2.95)	2.13 (1.58, 2.88)	2.05 (1.52, 2.76)
CP and LDR	[REDACTED]	5.66 (4.31, 7.44)	5.31 (4.04, 6.99)	4.94 (3.72, 6.55)	5.84 (4.45, 7.68)	5.1 (3.85, 6.75)
LDR with no DS or CP	[REDACTED]	3.09 (2.83, 3.38)	2.8 (2.55, 3.07)	2.82 (2.53, 3.13)	2.93 (2.68, 3.21)	2.57 (2.33, 2.83)

LDR Learning Disability Register; DS Down's syndrome; CP Cerebral Palsy

Table A5b. Estimated hazard ratio for non-COVID-19 related death in wave 2 (1 September 2020 – January 2021) in adults 16 and over

		Estimated Hazard Ratios (95% confidence intervals)				
Exposure category	*Events*	*Confounders*	*Confounders + IMD*	*Confounders + residential care*	*Confounders + comorbidities*	*All*
LDR						
No	58,021					
Yes	524	3.97 (3.65, 4.32)	3.63 (3.34, 3.95)	3.69 (3.34, 4.08)	3.73 (3.42, 4.06)	3.31 (3.02, 3.64)
by severity:						
Mild-moderate	392	3.60 (3.27, 3.97)	3.28 (2.97, 3.61)	3.42 (3.08, 3.81)	3.34 (3.03, 3.68)	3.02 (2.72, 3.34)
Severe-profound	132	5.70 (4.80, 6.76)	5.34 (4.50, 6.33)	5.11 (4.22, 6.19)	5.65 (4.77, 6.71)	5.02 (4.17, 6.04)
by residential care status:						
Not in residential care	397	3.83 (3.48, 4.21)	3.50 (3.19, 3.86)		3.58 (3.25, 3.94)	3.38 (3.07, 3.71)
In residential care	73	4.45 (3.51, 5.65)	4.16 (3.27, 5.29)		4.22 (3.32, 5.36)	1.67 (1.12, 2.5)
Down's syndrome						
No	58,482					
Yes	63	9.83 (7.53, 12.81)	9.45 (7.25, 12.33)	6.91 (5.27, 9.05)	6.61 (5.08, 8.58)	4.9 (3.72, 6.44)
Cerebral Palsy						
No	58,478					
Yes	67	2.64 (2.10, 3.33)	2.50 (1.98, 3.15)	2.24 (1.76, 2.85)	2.71 (2.14, 3.43)	2.33 (1.83, 2.97)
Combined grouping						
None	57,989					
DS but not LDR	0	-	-	-	-	-
DS and LDR	63	11.28 (8.68, 14.65)	10.86 (8.36, 14.11)	10.28 (7.9, 13.38)	7.26 (5.60, 9.42)	6.67 (5.12, 8.68)
CP but not LDR	32	1.78 (1.27, 2.50)	1.69 (1.21, 2.37)	1.78 (1.27, 2.50)	1.81 (1.29, 2.54)	1.73 (1.24, 2.43)
CP and LDR	35	4.92 (3.56, 6.80)	4.62 (3.33, 6.41)	4.47 (3.19, 6.27)	5.17 (3.72, 7.19)	4.62 (3.28, 6.49)
LDR with no DS or CP	426	3.58 (3.26, 3.92)	3.25 (2.97, 3.57)	3.36 (3.02, 3.74)	3.41 (3.10, 3.74)	3.05 (2.76, 3.37)

LDR Learning Disability Register; DS Down's syndrome; CP Cerebral Palsy;

Table A6. Baseline characteristics for adults and children among the whole sample, including those with missing ethnicity data

Characteristic	*Adults (16+ years)* On the learning disability register N (%)	Not on the learning disability register N (%)	*Children (<16 years)* On the learning disability register N (%)	Not on the learning disability register N (%)
Total	114,158 (100)	19,294,071 (100)	12,294 (100)	4,225,148 (100)
Age group				
0-15	0 (0)	0 (0)	12,294 (100)	4,225,148 (100)
16-44	68,988 (60)	8,755,777 (45)	0 (0)	0 (0)
45-64	34,470 (30)	6,128,473 (32)	0 (0)	0 (0)
65-69	4,605 (4)	1,176,842 (6)	0 (0)	0 (0)
70-74	3,259 (3)	1,187,836 (6)	0 (0)	0 (0)
75-79	1,679 (1)	843,729 (4)	0 (0)	0 (0)
≥80	1,157 (1)	1,201,414 (6)	0 (0)	0 (0)
Sex				
Female	45,450 (40)	9,702,494 (50)	3,967 (32)	2,061,624 (49)
Male	68,708 (60)	9,591,577 (50)	8,327 (68)	2,163,524 (51)
Ethnicity				
White	81,261 (71)	12,044,684 (62)	7,120 (58)	2,071,494 (49)
Black	1,710 (1)	402,235 (2)	379 (3)	96,181 (2)
South Asian	5,499 (5)	1,179,377 (6)	1,271 (10)	279,286 (7)
Mixed	1,125 (1)	206,860 (1)	307 (2)	99,517 (2)
Other	712 (1)	388,560 (2)	221 (2)	71,242 (2)
Missing	23,851 (21)	5,072,355 (26)	2,996 (24)	1,607,428 (38)
Region				
East	23,745 (21)	4,488,902 (23)	2,496 (20)	1,019,693 (24)
London	4,162 (4)	1,403,495 (7)	695 (6)	267,633 (6)
Midlands	25,764 (23)	4,149,948 (22)	2,923 (24)	943,910 (22)
North East, Yorks.*	24,450 (21)	3,597,648 (19)	2,709 (22)	837,543 (20)
North West	11,934 (10)	1,664,488 (9)	1,227 (10)	359,020 (8)
South East	7,467 (7)	1,299,204 (7)	762 (6)	258,290 (6)
South West	16,636 (15)	2,690,386 (14)	1,482 (12)	539,059 (13)
Index of multiple deprivation				
1 (Least deprived)	13,394 (12)	3,955,753 (21)	1,710 (14)	789,568 (19)
2	17,841 (16)	3,921,163 (20)	1,845 (15)	757,836 (18)
3	22,392 (20)	3,952,626 (20)	2,212 (18)	791,865 (19)
4	26,848 (24)	3,812,102 (20)	2,776 (23)	852,299 (20)
5 (Most deprived)	33,683 (30)	3,652,427 (19)	3,751 (31)	1,033,580 (24)
**Learning disability and related**				
Mild-moderate learning disability	94,248 (83)	-	10,497 (85)	-

Severe-profound learning disability	19,910 (17)	-	1,797 (15)	-
Not in residential care**	104,644 (92)	19,292,300 (100)	12,218 (99)	4,225,081 (100)
Residential care**	9,514 (8)	1,771 (<1)	76 (1)	67 (<1)
No Down's syndrome	105,027 (92)	19,292,656 (100)	10,977 (89)	4,222,216 (100)
Down's syndrome	9,131 (8)	1,415 (<1)	1,317 (11)	2,932 (<1)
No Cerebral Palsy	105,345 (92)	19,277,812 (100)	11,605 (94)	4,219,133 (100)
Cerebral Palsy	8,813 (8)	16,259 (<1)	689 (6)	6,015 (<1)
**Comorbidities**				
BMI≥40 (Obese III)	7,059 (6)	502,519 (3)	95 (1)	7,197 (0)
Asthma (with OCS use)	1,023 (1)	164,830 (1)		
Cystic fibrosis	42 (<1)	5,049 (<1)		
Respiratory disease	4,205 (4)	739,697 (4)		
Chronic cardiac disease	6,969 (6)	1,234,507 (6)		
Atrial fibrillation	2,914 (3)	703,140 (4)		
Deep vein thrombosis/ Pulmonary embolism	2,420 (2)	387,651 (2)		
Diabetes				
With HbA1c < 58 mmol mol−1	7,972 (7)	1,104,826 (6)		
With HbA1c ≥ 58 mmol mol−1	4,024 (4)	524,013 (3)		
With no recent HbA1c measure	1,604 (1)	210,645 (1)		
Liver disease	539 (<1)	108,517 (1)		
Stroke	2,356 (2)	389,337 (2)		
Transient Ischaemic Attack	1,111 (1)	298,222 (2)		
Dementia	2,252 (2)	226,508 (1)		
Other neurological disease	11,151 (10)	174,178 (1)		
Poor kidney function				
Stage 3a/3b, eGFR 30-60	3,440 (3)	995,610 (5)		
Stage 4/5, eGFR<30	635 (1)	95,155 (<1)		
Dysplenia	172 (<1)	30,052 (<1)		
Organ transplant	244 (<1)	17,928 (<1)		
Conditions leading to immunosuppression	609 (1)	50,411 (<1)		
Haematological malignancy				
Diagnosed last year	28 (<1)	9,611 (<1)		
Diagnosed 2-5 years ago	94 (<1)	29,438 (<1)		

Diagnosed >5 years ago	342 (<1)	67,283 (<1)
Cancer (non-haematological) in last year	230 (<1)	84,242 (<1)
RA/SLE/psoriasis	5,173 (5)	936,828 (5)
Inflammatory bowel disease	1,079 (1)	239,169 (1)
Serious mental illness	9,109 (8)	204,018 (1)

* North East, Yorkshire & The Humber; ** living in a household containing at least five individuals identified as being on the learning disability register

Table A7a. Estimated hazard ratio for COVID-19 related death and hospital admissions in waves 1 (1 March 2020 – 31 August 2020) and 2 (1 September 2020 – January 2021) in adults 16 and over, comparing the main analysis to one handling missing data in BMI using a complete case approach and applying multiple imputation to handle missing ethnicity data

		Estimated Hazard Ratios (95% confidence intervals)				
Outcome	*Exposure category*	*Confounders*	*Confounders + IMD*	*Confounders + residential care*	*Confounders + comorbidities*	*All*
Wave 1						
Covid-19 related hospital admission	Main analysis	5.30 (4.85, 5.80)	4.82 (4.41, 5.27)	4.70 (4.26, 5.18)	4.63 (4.24, 5.07)	3.92 (3.56, 4.30)
	Complete case for BMI	5.08 (4.64, 5.57)	4.62 (4.21, 5.06)	4.47 (4.04, 4.94)	4.45 (4.06, 4.88)	3.74 (3.39, 4.13)
	MI for ethnicity	5.33 (4.90, 5.79)	4.84 (4.45, 5.28)	4.70 (4.27, 5.17)	4.65 (4.28, 5.06)	3.92 (3.57, 4.30)
Covid-19 related death	Main analysis	8.21 (7.15, 9.42)	7.30 (6.35, 8.39)	6.96 (5.85, 8.28)	6.70 (5.81, 7.71)	5.58 (4.76, 6.53)
	Complete case for BMI	7.93 (6.88, 9.15)	7.04 (6.10, 8.13)	6.67 (5.56, 8.01)	6.48 (5.60, 7.50)	5.37 (4.55, 6.33)
	MI for ethnicity	8.11 (7.14, 9.22)	7.26 (6.37, 8.27)	6.72 (5.67, 7.97)	6.65 (5.84, 7.58)	5.47 (4.71, 6.35)
Wave 2						
Covid-19 related hospital admission	Main analysis	4.32 (4.05, 4.61)	3.91 (3.66, 4.18)	3.87 (3.59, 4.18)	3.8 (3.56, 4.05)	3.21 (2.98, 3.46)
	Complete case for BMI	4.12 (3.86, 4.41)	3.73 (3.48, 3.99)	3.68 (3.40, 3.98)	3.63 (3.4, 3.88)	3.06 (2.83, 3.30)
	MI for ethnicity	4.34 (4.07, 4.61)	3.94 (3.69, 4.21)	3.87 (3.58, 4.18)	3.82 (3.59, 4.06)	3.22 (2.99, 3.47)
Covid-19 related death	Main analysis	7.22 (6.41, 8.13)	6.39 (5.66, 7.22)	6.67 (5.86, 7.60)	6.31 (5.59, 7.11)	5.52 (4.85, 6.28)
	Complete case for BMI	7.03 (6.20, 7.96)	6.21 (5.46, 7.07)	6.54 (5.69, 7.52)	6.12 (5.40, 6.95)	5.40 (4.70, 6.20)
	MI for ethnicity	7.06 (6.26, 7.95)	6.27 (5.52, 7.11)	6.43 (5.62, 7.37)	6.18 (5.49, 6.96)	5.34 (4.68, 6.10)

LDR Learning Disability Register; DS Down’s syndrome; CP Cerebral Palsy; MI multiple imputation.

Table A7b. Estimated hazard ratio for COVID-19 related hospital admissions waves 1 (1 March 2020 – 31 August 2020) and 2 (1 September 2020 – January 2021) in children under 16, comparing the main analysis with one using multiple imputation for missing ethnicity

		Estimated Hazard Ratios (95% confidence intervals)				
Outcome	*Exposure category*	*Confounders*	*Confounders + IMD*	*Confounders + residential care*	*Confounders + comorbidities*	*All*
Wave 1						
Covid-19 related hospital admission	Main analysis	6.21 (2.75, 14.05)	6.13 (2.71, 13.90)	6.24 (2.76, 14.12)	6.25 (2.76, 14.13)	6.20 (2.74, 14.05)
	MI for ethnicity	4.77 (2.12, 10.73)	4.75 (2.11, 10.68)	4.78 (2.13, 10.77)	4.77 (2.12, 10.74)	4.77 (2.12, 10.74)
Wave 2						
Covid-19 related hospital admission	Main analysis	9.18 (5.89, 14.29)	8.98 (5.76, 13.98)	9.24 (5.93, 14.38)	8.96 (5.75, 13.97)	8.83 (5.67, 13.77)
	MI for ethnicity	9.71 (6.49, 14.52)	9.33 (6.24, 13.96)	9.76 (6.53, 14.60)	9.60 (6.42, 14.36)	9.29 (6.21, 13.89)

LDR Learning Disability Register; DS Down’s syndrome; CP Cerebral Palsy; MI multiple imputation.

Table A8. Codelists used to define variables used in the analysis

Variable	Notes	Codelist
On the learning disability register	Plus any individuals identified as severe and profound learning disability (all of whom should be on the learning disability register)	https://codelists.opensafely.org/codelist/opensafely/learning-disabilities/2020-07-06/
Severe and profound learning disability		https://codelists.opensafely.org/codelist/opensafely/severe-and-profound-learning-disability-flags/44ef542a/#full-list
Down's syndrome		https://codelists.opensafely.org/codelist/opensafely/down-syndrome/15832db6/#full-list
Cerebral Palsy		https://codelists.opensafely.org/codelist/opensafely/cerebral-palsy/1835edac/#full-list
Ethnicity	5 categories, obtained from 16 (White, South Asian, Black, Mixed, Other)	https://codelists.opensafely.org/codelist/opensafely/ethnicity
Chronic cardiac disease		https://codelists.opensafely.org/codelist/opensafely/chronic-cardiac-disease/
Atrial Fibrillation	This codelist includes both atrial fibrillation and atrial flutter	https://codelists.opensafely.org/codelist/opensafely/atrial-fibrillation-or-flutter/2020-07-30/
Prior deep vein thrombosis / pulmonary embolism		https://codelists.opensafely.org/codelist/opensafely/venous-thromboembolic-disease/2020-09-14/

Diabetes	Combined with Hba1c measure within 18 months to determine level of control	https://codelists.opensafely.org/codelist/opensafely/diabetes/
Stroke and transient ischaemic attack		https://codelists.opensafely.org/codelist/opensafely/stroke-updated/2020-06-02/ https://codelists.opensafely.org/codelist/opensafely/transient-ischaemic-attack/3526e2ac/
Dementia		https://codelists.opensafely.org/codelist/opensafely/dementia-complete/48c76cf8
Other neurological conditions	Will be used only for exclusion of individuals eligible for vaccination in final analysis: will not be adjusted for in models.	https://codelists.opensafely.org/codelist/opensafely/other-neurological-conditions/
Asthma	Combined with OCS prescriptions in past year to determine severity	https://codelists.opensafely.org/codelist/opensafely/asthma-diagnosis/
Cystic Fibrosis and associated conditions		https://codelists.opensafely.org/codelist/opensafely/cystic-fibrosis/2020-07-20/
Respiratory disease other than asthma or cystic fibrosis		https://codelists.opensafely.org/codelist/opensafely/other-chronic-respiratory-disease/2020-07-20/
Non-haematological cancer	Incident diagnosis within the previous year	https://codelists.opensafely.org/codelist/opensafely/cancer-excluding-lung-and-haematological/
Haematological cancer	Grouped by time since diagnosis (<1 year, 2-<5 years, 5+years)	https://codelists.opensafely.org/codelist/opensafely/haematological-cancer/

Lung cancer	Incident diagnosis within the previous year, combined with other non-haematological cancer	https://codelists.opensafely.org/codelist/opensafely/lung-cancer/
Liver disease		https://codelists.opensafely.org/codelist/opensafely/chronic-liver-disease/
Kidney dialysis	Used if no kidney transplant since most recent dialysis	https://codelists.opensafely.org/codelist/opensafely/dialysis/2020-07-16/
Kidney transplant	Combined with non-kidney transplant for transplant indicator. Also used to determine which of dialysis/transplant is most recent.	https://codelists.opensafely.org/codelist/opensafely/kidney-transplant/2020-07-15/
Organ transplant (other than kidney)	Combined with kidney transplant for transplant indicator.	https://codelists.opensafely.org/codelist/opensafely/other-organ-transplant/2020-07-15/
Asplenia		https://codelists.opensafely.org/codelist/opensafely/asplenia/ https://codelists.opensafely.org/codelist/opensafely/sickle-cell-disease/
Rheumatoid arthritis, lupus, psoriasis		https://codelists.opensafely.org/codelist/opensafely/ra-sle-psoriasis/
Other immunosuppressive condition	Temporary and aplastic anaemia within last year; HIV and other permanent immunosuppression ever.	https://codelists.opensafely.org/codelist/opensafely/hiv/2020-07-13/ https://codelists.opensafely.org/codelist/opensafely/permanent-immunosuppresion/ https://codelists.opensafely.org/codelist/opensafely/aplastic-anaemia/ https://codelists.opensafely.org/codelist/opensafely/temporary-immunosuppresion/
Inflammatory bowel disease		https://codelists.opensafely.org/codelist/opensafely/inflammatory-bowel-disease/2020-04-07/

Serious mental illness	Psychosis, schizophrenia and bipolar affective disease	https://codelists.opensafely.org/codelist/opensafely/psychosis-schizophrenia-bipolar-affective-disease/2020-07-09/
